# OMAnnotator: a novel approach to building an annotated consensus genome sequence

**DOI:** 10.1093/bioadv/vbag015

**Published:** 2026-01-22

**Authors:** Sadé Bates, Christophe Dessimoz, Yannis Nevers

**Affiliations:** Department of Computational Biology, University of Lausanne, CH-1015 Lausanne, Switzerland; Department of Genetics, Evolution and Environment, University College London, London WC1E 6AA, United Kingdom; Department of Integrative Biology, University of California, Berkeley, CA 94720, United States; Department of Computational Biology, University of Lausanne, CH-1015 Lausanne, Switzerland; Department of Genetics, Evolution and Environment, University College London, London WC1E 6AA, United Kingdom; Department of Computational Biology, University of Lausanne, CH-1015 Lausanne, Switzerland; Complex Systems and Translational Bioinformatics (CSTB), Department of Computer Science, UMR7357 ICube, University of Strasbourg, CNRS, Centre de Recherche en Biomédecine de Strasbourg, 67000 Strasbourg, France

## Abstract

**Motivation:**

Advances in sequencing technologies have enabled researchers to sequence whole genomes rapidly and cheaply. However, despite improvements in genome assembly, structural genome annotation (i.e. the identification of protein-coding genes) remains challenging, particularly for eukaryotic genomes. It requires using several approaches (typically *ab initio*, transcriptomics, and homology search), which may give substantially different results. Deciding which gene models to retain in a consensus is far from trivial, and automated approaches tend to lag behind laborious manual curation efforts in accuracy.

**Results:**

We present OMAnnotator, a novel approach to building a consensus annotation. OMAnnotator repurposes the OMA algorithm, originally designed to elucidate evolutionary relationships among genes across species, to integrate predictions from different annotation sources into a consensus annotation, using evolutionary information as a tie-breaker. During benchmarking on the *Drosophila melanogaster* reference, OMAnnotator’s consensus improved upon its source annotations and two state-of-the-art pipelines used as annotation combiners with the same inputs. When applied to three recently published genomes, OMAnnotator gave substantial improvements in two cases, and mixed results in the third, which had already benefitted from extensive expert curation. This underlines the method’s effectiveness and robustness for combining the results of disagreeing annotation softwares, strengthening the toolkit for eukaryotic genome annotation.

**Availability and implementation:**

OMAnnotator is available on GitHub (https://github.com/DessimozLab/OMAnnotator).

## 1 Introduction

With the advances in sequencing technology, sequencing a genome is faster and more affordable than ever. However, annotating the increasing number of newly sequenced genomes remains labour-intensive and error-prone (Salzberg 2019, Meyer *et al.* 2020, Scalzitti *et al.* 2020). Genome annotation involves identifying the features in a genome sequence, including protein coding or non-coding genes, regulatory elements and structural variants such as inversions and repeats, which is essential for understanding its underlying biology. Here, we focus on the prediction of protein coding genes and describe a new approach to improve the ease and accuracy of this process.

Three major classes of methods are used to predict genes in genome assemblies: *ab initio* gene prediction, transcript alignment and homology alignment (Hoff and Stanke 2015, Mudge and Harrow 2016). The first of these involves using a gene finder algorithm, such as AUGUSTUS (Stanke *et al.* 2008), to identify genes in a genome assembly based on statistical models trained on known gene structures, for example in model species. Transcript alignment is the alignment of reads from RNA sequencing to the reference assembly to locate transcribed regions of the genomes. Finally, homology alignment involves searching for regions in the newly sequenced genomes that are similar to coding genes in a closely related species to identify likely homologous genes.

One of the main challenges in genome annotation is combining the genes predicted by different—and sometimes contradictory—annotation methods into a consensus. A good consensus annotation accurately captures most genes in the genome, i.e. it retains the maximum number of true gene predictions while dropping false gene predictions. Genome annotation pipelines such as BRAKER (Brůna *et al.* 2021, Gabriel *et al.* 2024) aim to achieve this using RNAseq or protein sequence evidence during the AUGUSTUS (Stanke *et al.* 2008) iterative training and gene prediction processes, which improves annotation accuracy compared to using AUGUSTUS alone. Nevertheless, combining gene models from multiple evidence sources tends to be needed to remove false predictions from the consensus annotation. Existing approaches such as EVidence Modeller (EVM) (Haas *et al.* 2008) produce a consensus annotation by assigning quality weightings to annotation sets produced by different annotation methods. Although this increases the specificity of the annotation by dropping false predictions from a lower-quality set, less abundant true gene predictions may also be excluded from the consensus.

**Figure 1:**
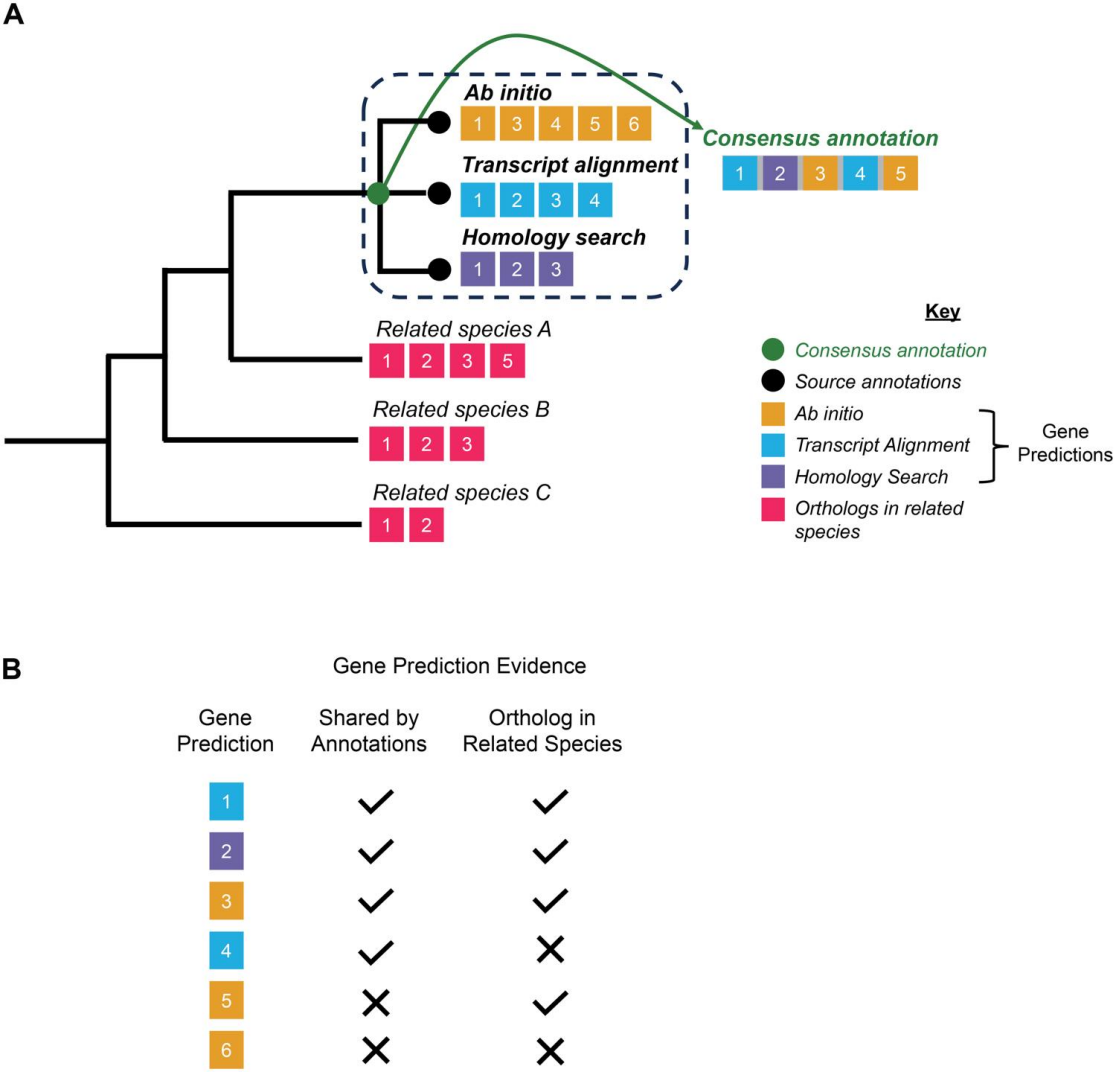
Overview of the OMAnnotator approach. (A) An example of a simple input species tree is shown on the left. The numbered squares inside the dashed box represent gene predictions from source annotations, and the numbered squares outside the box represent their orthologs in related species. OMAnnotator combines predictions from individual source annotations into a consensus, leveraging orthology evidence from related species where gene predictions are not shared between annotations. (B) Summary of evidence for retaining gene predictions. The colour of the gene prediction reflects its source annotation. OMAnnotator retains gene predictions (numbered coloured squares) that are shared by the annotation sets (predictions 1–4), or have an orthologue in a related species (predictions 1–3 and 5). Where gene predictions are shared between annotations, the predictions with the longest sequence are retained by default.

To approach this long-standing issue from a new angle, we sought to build consensus gene sets using an approach developed to model genome evolution. Our tool ‘OMAnnotator’ repurposes OMA (Orthologous MAtrix) standalone (Altenhoff *et al.* 2019, 2021), a state-of-the-art orthology inference software, to reconstruct a consensus annotation set from an arbitrary number of input annotation sets. OMA standalone infers Hierarchical Orthologous Groups (HOGs), sets of genes within a taxonomic range that descended from the same gene in the common ancestor of the taxon (Train *et al.* 2017, Zahn-Zabal *et al.* 2020). OMAnnotator combines annotations from different sources through its HOG inference software as if they were closely related species, with the consensus annotation being equivalent to the common ancestor of the annotation sets. By doing this, it selects gene models supported by multiple lines of evidence: agreement between predictions from the user-provided annotation sets (related by sequence similarity) and the existing annotations of related species (Fig. 1A). All genes retained in the consensus are supported by at least two lines of evidence (prediction by one annotation method as well as orthology with another annotation method and/or other species) (Fig. 1B).

We validated this approach by using it to re-annotate the latest *Drosophila melanogaster* genome assembly, starting from the ground up. OMAnnotator integrated three lines of evidence: *de novo* gene prediction, RNAseq data, and homology search. OMAnnotator’s output was then compared to the high-quality reference annotation available for this model organism. We also compared the OMAnnotator results to those obtained using EVM (Haas *et al.* 2008) and BRAKER (Brůna *et al.* 2021, Gabriel *et al.* 2024) on the same input data. Finally, we used OMAnnotator to re-annotate the genomes of three species previously annotated with EVM (Haas *et al.* 2008).

## 2 Methods

### 2.1 Description of the OMAnnotator pipeline

**Figure 2:**
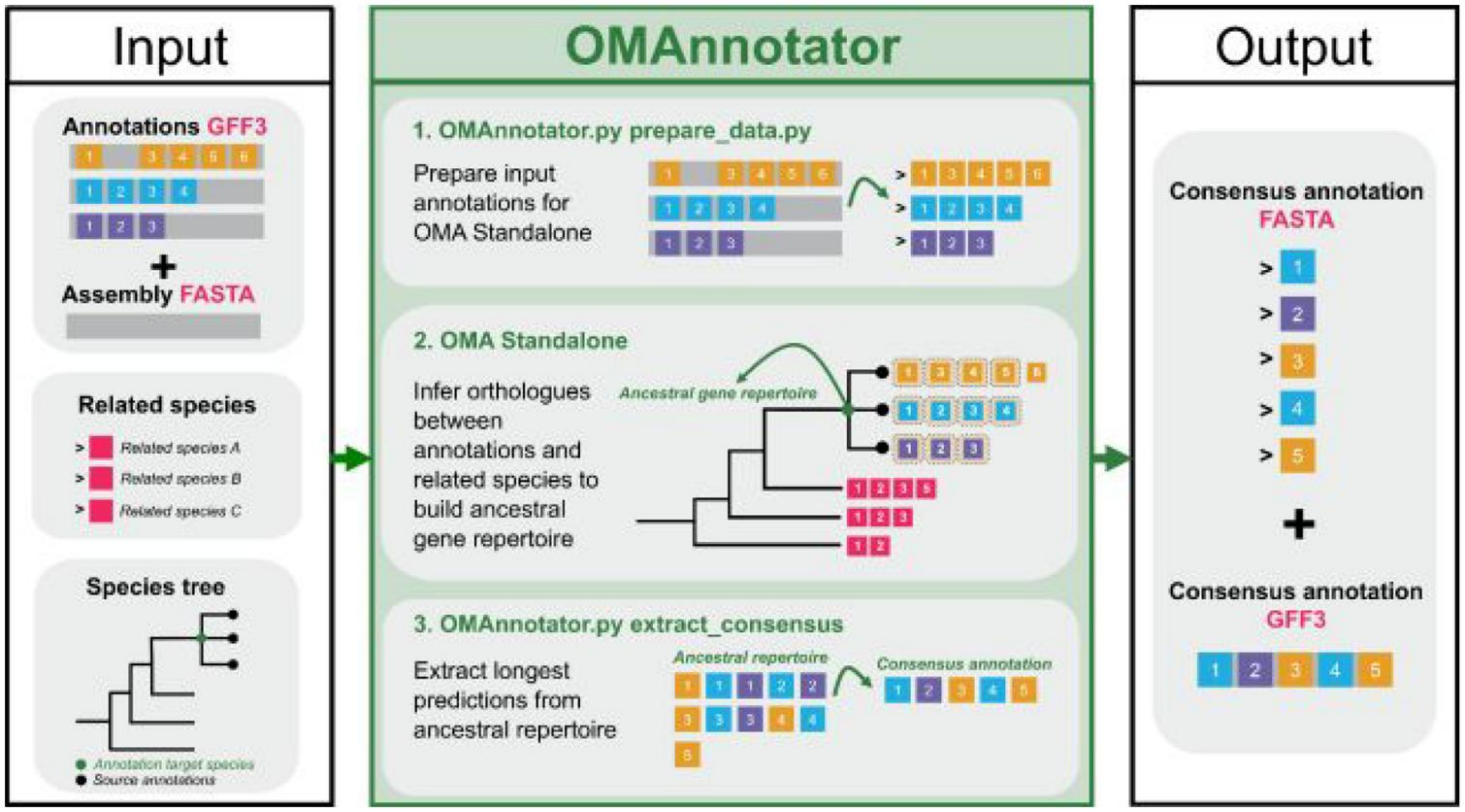
An overview of the OMAnnotator workflow. OMAnnotator takes as input: GFF3 format annotations from different sources, a FASTA format genome assembly, and a file of precomputed orthology relationships between a set of user-selected species that are related to the annotation target. This ‘related species’ file can be downloaded from the OMA browser. (1) The ‘prepare_data’ command from OMAnnotator.py prepares the input data for OMA Standalone. **(**2) The user-specified species tree is added to the OMA Standalone parameters file. OMA Standalone infers orthology between the annotations and related species at the ancestral node. (3) Gene predictions that are supported by multiple annotations and/or have an orthologue in a related species are extracted using the ‘extract_consensus’ command from OMAnnotator.py. Where gene predictions are shared between annotations, the prediction with the longest CDS is selected as representative in the consensus annotation by default. OMAnnotator outputs the consensus annotation in FASTA and GFF3 formats.

The OMAnnotator pipeline relies on the inference of orthologous groups provided by the OMA Standalone software (Altenhoff *et al.* 2019, 2021) to combine gene predictions from different annotation methods into a consensus annotation. The pipeline takes GFF3 files produced by any annotation methods as its primary input, uses them to form a consensus annotation and outputs a combined annotation in GFF3 and FASTA format. It is executed in three main steps: setting up annotation data, HOG inference, and consensus annotation extraction (Fig. 2 for an overview).

#### 2.1.1 Setting up annotation data for OMAnnotator

A local copy of the OMA Standalone software and a file of precomputed orthology relationships between a set of species are prerequisites. We recommend the species be selected to maximize taxonomic diversity and include species that are closely related to the species being annotated. Such a file can be downloaded from the ‘Download>Export All-All’ section of the OMA Browser (https://omabrowser.org/oma/export/), which downloads an archive of precomputed pairwise sequence comparisons between the proteins of the selected species. Both the OMA Standalone software and the precomputed orthology relationships are locally stored in what will be hereafter referred to as the ‘OMA folder’.

The first step of the pipeline involves extracting the information needed to run the OMA algorithm from the input GFF3 files and the target species’ genomic sequence (Fig. 2). This process is automated through the ‘prepare_data’ command of the OMAnnotator software, which takes as input a folder containing any number of GFF3 annotation files and the genome assembly sequence to which they correspond. As feature naming by different annotation software often deviates from the GFF3 specifications (Stein 2020), an optional ‘feature_type’ argument to our prepare_data function allows OMAnnotator to adapt to different denominations for genomic elements. This argument takes a tab-separated value file indicating which features in the annotation file correspond to a gene, transcript or CDS according to the GFF3 specifications, giving OMAnnotator the flexibility to process annotations from a variety of software without the need for the user to reformat their source annotations. The prepare_data command extracts all protein sequences described in a GFF3 file and adds its resulting FASTA file to the ‘DB’ (database) subfolder in the user’s OMA folder. If any gene is predicted to have multiple isoforms, alternative splicing information is added to the DB subfolder as a ‘Splice file’. This enables OMA Standalone to select the isoform sequence most similar to its detected homologues as the main representative of each gene in its consensus estimation. All isoforms for the selected gene will nonetheless be added to the final consensus annotation. The prepare_data command also writes a species tree file for the user-selected related species to the parameters file in the OMA folder.

#### 2.1.2 OMA standalone

Next, the OMA Standalone pipeline is run as described in Altenhoff *et al.* (2019, 2021) to infer orthology relationships between proteomes. For this purpose, the parameter file is edited to specify a species tree (in Newick format) that includes the various input annotation sets to be combined, and the user-selected related species. We recommend that users edit the Newick format string that the prepare_data command writes to the parameter file by adding a node corresponding to the species, whose children leaves are the source annotations. The leaves descending from this node must share the same names as the annotation FASTA files in the DB folder.

#### 2.1.3 Consensus extraction

The last step of the pipeline is the extraction of a consensus annotation from the OMA Standalone output. This is done using the ‘extract_consensus’ command of the OMAnnotator software, which takes as input the species tree specified above and the HierarchicalGroups.orthoxml file of HOGs outputted by OMA Standalone. It generates a protein FASTA file and a GFF3 file corresponding to the consensus annotation.

The software retains as consensus genes any gene that is present in the OMA inferred ‘ancestral genome’ of the different annotation methods. As such, the ancestral genome contains any sequence shared by at least two annotation methods or those predicted in one of the annotations as long as they have detected orthologs in any outgroup species. This allows combining genes inferred by multiple methods but disregarding the ones with low support. When multiple annotation methods predict a gene, the version with the longest coding sequence is selected as the representative sequence for the consensus set as default. However, users can provide a list of sources ranked by expected quality of gene models with the ‘—priority’ (-p) option. If this option is used, OMAnnotator will always provide the gene model from the highest ranking source in order of priority. If a selected gene model has multiple transcripts in its source annotation, all its isoforms will be included in the final annotation.

OMAnnotator also provides two text files as reports of the final annotation. The first report, with the suffix ‘_report.txt’ is a text file summarizing how many genes were retained in the final annotation, how many genes were supported by any combination of sources, and finally how many times each source annotation was used as the source of gene models. The second report, with the suffix ‘_detailed_report.txt’ is a tab separated file, where each line corresponds to a gene in the final annotation, and each column corresponds to source of support for the predicted gene—either a source annotation or any external species. This file details, as a binary matrix, which source or species did or did not support each of the selected genes.

### 2.2 Proof of principle: annotating the *D. melanogaster* genome sequence

To validate the OMAnnotator approach, we used it to annotate the *D. melanogaster* genome sequence from scratch. We downloaded the latest genome assembly (genomic release 6.32) without annotations from FlyBase and used three annotation methods to predict genes. The resulting annotation sets into a single FASTA and GFF3 sequence file using OMAnnotator. We measured OMAnnotator compute resource requirements using various numbers of input annotations and species in the precomputed species set. Finally, we compared OMAnnotator’s results to those of the BRAKER (Brůna *et al.* 2021, Gabriel *et al.* 2024) and EVM (Haas *et al.* 2008) pipelines, using the same input data.

#### 2.2.1 Gene prediction using AUGUSTUS

We ran AUGUSTUS v3.2.3 (Stanke *et al.* 2008) with the unannotated *D. melanogaster* genome as input and the ‘—species=fly’ option to specify *D. melanogaster* gene model parameters. The ‘—gff3’ output format flag was set to produce an annotation in AUGUSTUS’ GFF3 format. This file was reformatted to meet GFF3 standards (Stein 2020) for downstream analyses using the custom script ‘reformat_augustus_gff3_output.sh’ (all custom scripts/python notebooks used in this study are available from the archive at https://doi.org/10.5281/zenodo.14252311).

#### 2.2.2 Transcript alignment using StringTie

Seventeen-day *D. melanogaster* adult tissue RNAseq data generated with paired-end Illumina sequencing during a study of the *D. melanogaster* developmental transcriptome (Daines *et al.* 2011) were downloaded from NCBI SRA (accession number SRS065821). Sequences were joined in FASTQ format using the fastq-dump tool from the SRA toolkit v2.10.9, and their quality was checked using FastQC (Andrews 2010). The adapter sequences were then trimmed using Trimmomatic v0.39 (Bolger *et al.* 2014) in Paired End mode with the parameters ‘-phred33 -threads 24 ILLUMINACLIP: RNAseq_data/TruSeq2-PE.fa:2:30:10 LEADING:3 TRAILING:3 SLIDINGWINDOW:4:15 MINLEN:36’. Next, reads were aligned to the reference genome using HISAT2 v2.2.0 (Kim *et al.* 2015), producing an alignment BAM file. Transcripts were assembled from this alignment file using StringTie v2.1.7 (Pertea *et al.* 2015), and the longest open reading frames were identified using TransDecoder v1 (Haas 2013), producing a GFF3 file of likely peptide sequences.

#### 2.2.3 Homology search using GeMoMa

*Anopheles gambiae* proteome data from the assembly AgamP4 (Sharakhova *et al.* 2007) was downloaded from UniProt on 25/10/2021. GeMoMa v1.7.1 (Keilwagen *et al.* 2019) was used to infer *D. melanogaster* gene models based on the *A. gambiae* protein sequences, with the following options specified ‘-Xmx50g GeMoMaPipeline GeMoMa.Score=ReAlign AnnotationFinalizer.r=NO o=true’.

#### 2.2.4 OMAnnotator: using OMA standalone orthology inference to form a consensus annotation

A file of precomputed orthology relationships between 25 species was downloaded from OMA Browser as described above. FASTA format sequence files and any accompanying splice files were produced using the prepare_data command of the OMAnnotator software as described above. A species tree of the 25 outgroup species and the three annotations clustered on a single branch within the *Drosophila* clade (Fig. 5, available as supplementary data at *Bioinformatics Advances* online) was specified as a Newick format species tree in OMA Standalone’s parameters file. OMA Standalone was then run as described above, and a consensus annotation set was produced using the ‘extract_consensus’ command of the OMAnnotator software.

#### 2.2.5 OMAnnotator compute resource requirements

**Figure 3:**
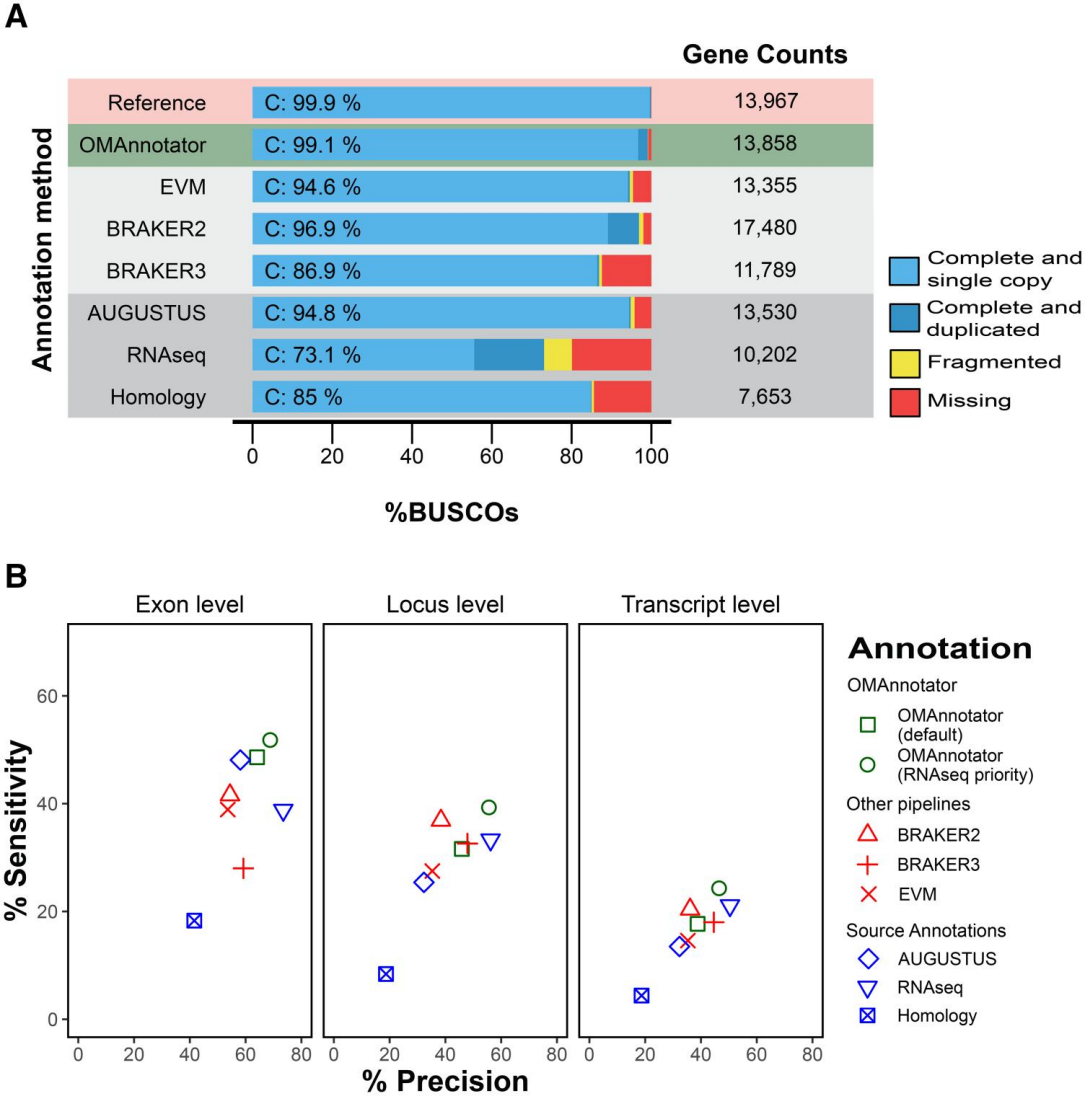
Proof of principle annotation quality results. (A) BUSCO analyses results and gene counts. The gold standard reference annotation (FlyBase genomic release 6.32, version 4) is is shown in the first row, followed by the consensus produced by OMAnnotator, cononsensus annotations produced by other piplelines, and the individual source annotations. (B) GffCompare analyses results. % Sensitivity and % Precision scores for each annotation are reported at the exon, gene locus and transcript levels. OMAnnotator (default) is the output from default OMAnnotator settings and OMAnnotator (RNAseq priority) is the output from setting priority for retaining models from the RNAseq source.

**Figure 4:**
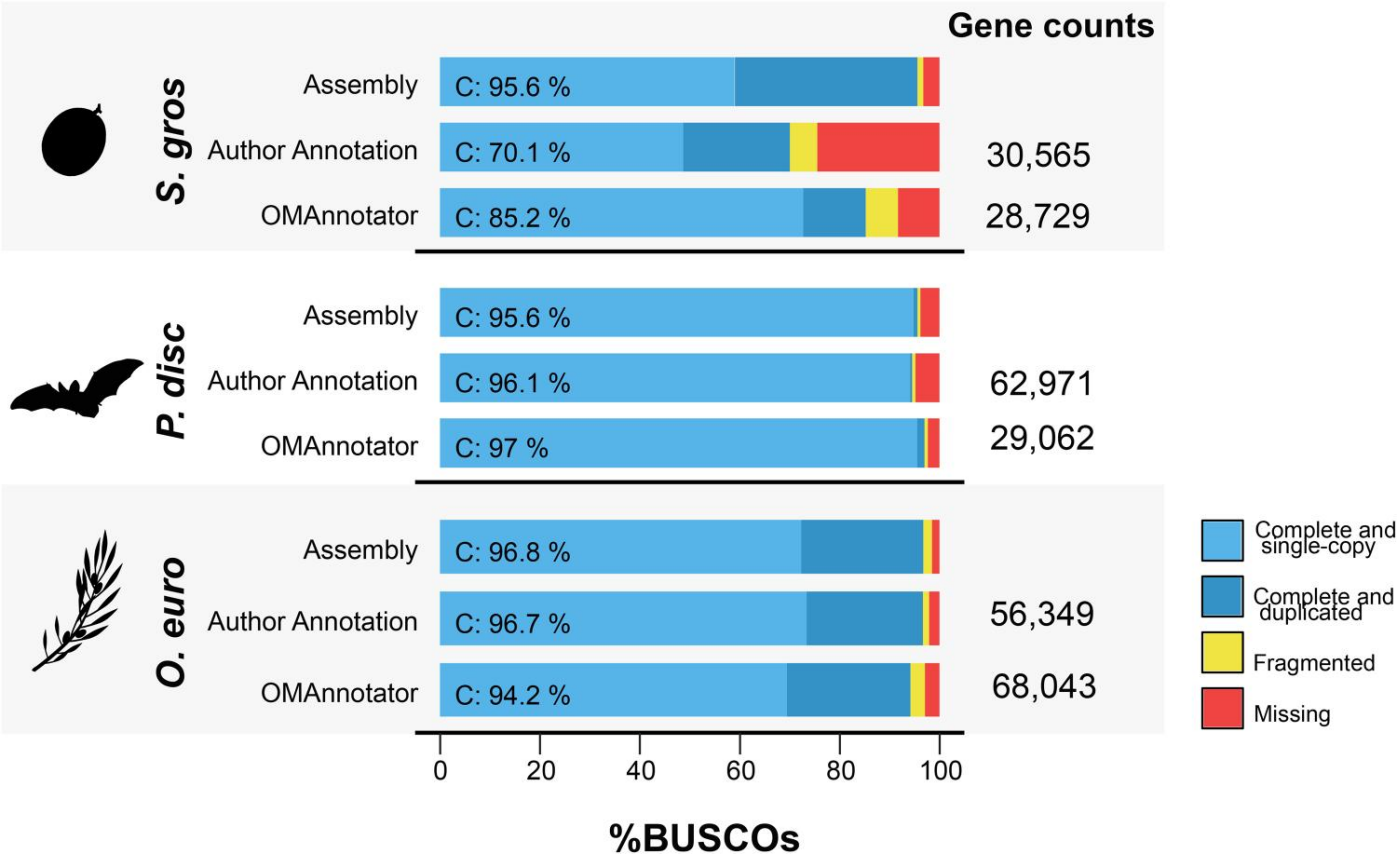
BUSCO results from using OMAnnotator to re-annotate three genomes: *Siratitia grosvenorii* (top row), *Phyllostomus* discolour (middle row) and *Olea europaea* (bottom row). The OMAnnotator gene set BUSCO scores are compared to the authors’ annotation and the genome assembly. Gene counts of the number of genes predicted by each annotation method are shown on the right.

Two runtime experiments were conducted to assess OMAnnotator’s required CPU hours and maximum RSS usage. First, OMAnnotator was run with the three source annotations and varying numbers of precomputed species (5, 10, 15, 20, and 25 species; see Figs. 1–5, available as supplementary data at *Bioinformatics Advances* online, for species trees) to determine the optimum number of input species (runtime versus consensus quality). Consensus annotation quality was assessed by calculating gene count, BUSCO completeness and GffCompare statistics (see Section 2.2.7 for detailed annotation quality analysis method). Second, OMAnnotator was run with the 10 species set (Fig. 2, available as supplementary data at *Bioinformatics Advances* online) from Experiment 1 and an increasing number of source annotation inputs (2, 3, 6, and 12 annotations), mimicking the situation where multiple lines of experimental data are available for the target species. The number of source annotations was increased by duplicating the RNAseq source annotation, from absent to 10× duplication. All runs were completed on the UNIL Curnagl infrastructure, a 96-node HPC cluster based on AMD Zen2/3 CPUs providing a total of 4608 compute cores and 54TB of memory. The three main stages of each OMA Standalone orthology inference step were executed using a SLURM scheduler with the following allocations: (i) 1 CPU, 50G memory; (ii) 500 parallelized jobs with 1 CPU and 4G memory each; (iii) 24 CPU, 40G memory. Resource usage was recorded from the SLURM efficiency (seff) command output. OMAnonator steps 1 and 3 were not measured, as unlike OMA Standalone, these steps do not need to be run on a HPC.

#### 2.2.6 Comparing OMAnnotator with other annotation pipelines

To benchmark the performance of OMAnnotator against other consensus approaches, we compared its output with that of EVM and BRAKER versions 2 and 3 (Haas *et al.* 2008, Brůna *et al.* 2021, Gabriel *et al.* 2024) when annotating the same *D. melanogaster* genome sequence using the same source annotations as inputs.

We reformatted the AUGUSTUS and homology annotations into GFF3 format required by EVM, using its in-built scripts ‘augustus_GFF3_to_EVM_GFF3’ and ‘GeMoMa_gff_to_gff3’. The RNAseq annotation did not require reformatting. We assigned each annotation a weight of ‘1’, as we had no evidence to suggest the quality of gene structures predicted varied consistently across annotation sources. The software was run with the parameters ‘–segmentSize <100000>’ and ‘–overlapSize <15000>’.

We then performed gene annotation with BRAKER, which has various modes for training gene predictors on different annotation inputs. We first ran BRAKER2 in three modes: with RNAseq alignments from the RNAseq source annotation, with the homology source annotation, and with a combination of the two. Finally BRAKER3 was run with the RNAseq data and homology annotation as inputs. Before performing quality assessments, we converted the BRAKER2 and BRAKER3 GTF output into GFF3 format using the ‘gtf_to_gff3.pl’ script from the GenomeTools package (Gremme *et al.* 2013), as its GFF3 files did not conform to GFF3 standards (Stein 2020). Where necessary, redundant sequences were removed using Awk. Prior to BUSCO analyses, the custom script ‘extract_pep.py’ and python notebooks were used to convert the reformatted GFF3 files into FASTA format and select the longest isoform per gene.

#### 2.2.7 Analysing the quality of annotations

All annotations were compared to the *D. melanogaster* reference annotation genomic release 6.32 downloaded from FlyBase (Gramates *et al.* 2022). The accuracy and completeness of gene content was assessed by gene count comparisons and BUSCO v5.4.2 analyses (Manni *et al.* 2021) using the *Dipteran* lineage set from the odb10 release.

The quality of the gene structure annotations in each annotation set were compared to the *D. melanogaster* reference annotation using GffCompare with the option ‘-D’ to discard duplicate query transcripts (Pertea and Pertea 2020). As the original *D. melanogaster* reference annotation had incompatibilities with GFF software due to the presence of a trans-spliced gene, the entries relative to this single gene were removed from the GFF3 annotation before any GFF comparison. BRAKER GFFs were reformatted using custom python notebooks. Once all annotations met GFF3 specifications, GffCompare was used to compute the Sensitivity and Precision scores at the exon, loci and transcript levels. According to GffCompare comparison rules (Pertea and Pertea 2020), an exon-level match (True Positive; TP) was counted if the boundaries of a predicted exon matched the reference. A locus-level TP was counted if at least one predicted transcript had a transcript level match with a reference transcript in the corresponding reference locus. Lastly, a transcript-level TP was counted if all internal exons in the exon chain matched, and the outer boundaries of the terminal query exons matched with at most 100 bases difference. Single exon transcripts were classed as a TP if they overlapped the reference by 80%.

### 2.3 Using OMAnnotator to re-annotate other genomes

To evaluate the flexibility and robustness of OMAnnotator on other genomes, we used it to re-annotate three non-model organism genomes for which the intermediate annotation data had been made available: *Siraitia grosvenorii* (Monk Fruit) (Xia *et al.* 2018), *Phyllostomus discolor* (Pale Spear-Nosed Bat) (Jebb *et al.* 2020), and *Olea europaea* (European Olive Tree) (Cruz *et al.* 2016). The genomes of each of these organisms were originally annotated using EVM (Haas *et al.* 2008) to combine source annotation sets. The authors of the original annotations kindly provided their input source annotations and the raw consensus output from EVM where available, else they provided their curated final consensus annotation.

For each re-annotation, 10 related species with good-quality proteomes were selected to maximize coverage of orders around the target species. OMAnnotator was then run with the GFF3 source annotations and genome assembly as input, with the species tree specified in the OMA Standalone parameter file (Figs 8–10, available as supplementary data at *Bioinformatics Advances* online). Finally, the OMAnnotator consensus annotations were compared with the authors’ sequences using gene counts and BUSCO (v5.4.2) analyses with the lineages eudicots_odb10 for *S. grosvenorii* and *O. europaea*, and laurasiatheria_odb10 for *P. discolor*. As these species are non-model organisms, BUSCO scores from each annotation method were compared with the genome assembly BUSCO scores instead of a reference annotation.

## 3 Results

### 3.1 Proof of principle

As a proof of principle, we used OMAnnotator to annotate the *D. melanogaster* reference genome assembly (FlyBase, Genomic Release 6.32) from scratch, with three source annotations generated using three primary annotation approaches: gene prediction with AUGUSTUS (Stanke *et al.* 2008), RNAseq transcript alignment with StringTie (Pertea *et al.* 2015), and homology search with GeMoMa (Keilwagen *et al.* 2019). We then compared all annotations to the *D. melanogaster* reference annotation (genomic release 6.32), which as one of the best annotated model organism genomes, serves as a gold standard. This *D. melanogaster* reference gene set contained 13,967 protein coding genes (Table 3, available as supplementary data at *Bioinformatics Advances* online, for more detailed gene content statistics) and 99.9% complete *Dipertan* BUSCOs (release odb10), of which a minimal fraction of 0.3% were duplicated (Fig. 3A).

#### 3.1.1 OMAnnotator performs better than individual methods

Of the source annotations, the homology annotation predicted the fewest genes at 7,592 and, accordingly, had a high percentage of missing BUSCOs (14.4%; Supplementary table 1) and the lowest sensitivity and precision scores across exon, locus and transcript feature levels (Supplementary table 2). The RNAseq annotation had highert number of predicted genes (10,202) but nevertheless had a high number of missing BUSCOs (19.9%) (Supplementary table 1). Its precision scores were higher than the other methods at the exon (73.5%), locus (56.2%), and transcript (50.4%) feature levels (Supplementary table 2). In contrast, the AUGUSTUS annotation gene count was closest to the reference, with 13,530 genes, and the lowest (though still notable) proportion of fragmented and missing BUSCO genes (1.0% fragmented, 4.2% missing) (Supplementary table 1). Accordingly, it had higher sensitivity and precision scores than the homology annotation across all feature levels, but lower scores than the RNAseq annotation at the locus and transcript levels (Fig. 3B).

The OMAnnotator consensus gene set [OMAnnotator (default)] was obtained by a consensus of the three annotations described above combined with homology data from 25 species. 8,939 predictions were retained from the AUGUSTUS source, 3,124 from the RNAseq source and 1,795 from the homology source. It was a clear improvement on its source annotations, with fewer missing genes and more complete BUSCOs (99.1%). It also had higher sensitivity and precision scores than the AUGUSTUS and homology annotations, but not the RNASeq annotation (Fig. 3B). We performed the same OMAnnotator run with the ‘—priority’ option set to prioritize the RNAseq source annotation when multiple sources were available for the same gene [OMAnnotator (RNAseq priority)] and improved its sensitivity and precision scores, particularly at the locus and transcript levels (Fig. 3B). This run retained 8,242 RNAseq predictions, 4,858 AUGUSTUS predictions, and 758 homology predictions.

#### 3.1.2 OMAnnotator compute resource requirements

To assess OMAnnotator compute requirements, we ran the software with varying sizes of precomputed species sets (5, 10, 15, 20 and 25 species; see Figs 1–5, available as supplementary data at *Bioinformatics Advances* online, for species trees) and varying numbers of source annotations (2, 3, 6, and 12 annotations). Only the OMA Standalone step of OMAnonator was measured, as this needs to be parallelized and run on an HPC. OMA Standalone required 281 total CPU hours and a maximum RSS of 2500M with 10 input species and three source annotations (Fig. 6A, available as supplementary data at *Bioinformatics Advances* online). Runtime and RSS requirements were dominated by the ‘allvsall’ alignment stage of OMA Standalone. The maximum RSS did not exceed 8000M, even with 25 input species. Increasing the number of species increased the resource usage, but any improvement in consensus annotation quality was marginal beyond 10 species (Fig. 6B and C, available as supplementary data at *Bioinformatics Advances* online). Thus, we settled on 10 species as our recommendation for future OMAnnotator runs.

#### 3.1.3 OMAnnotator performance improves upon other state-of-the-art pipelines

To compare OMAnnotator with state-of-the-art automated methods, we used two other annotation methods to annotate the *D. melanogaster* genome assembly, with different combinations of source annotations supplied as extrinsic evidence depending on the method’s requirements.

The first method, EVM, combines predictions from different sources using weights based on the abundance and source of each prediction (Haas *et al.* 2008). Using the AUGUSTUS, RNAseq and homology annotations as inputs resulted in a consensus annotation with 13,355 genes and 94.6% complete BUSCOs (Fig. 3A). While these scores indicate a high similarity to the reference annotation, its GffCompare sensitivity and precision scores were lower at the locus and transcript levels than OMAnnotator and BRAKER annotations (Fig. 3B).

We also ran BRAKER (2 and 3), which trains the gene finders GeneMark and AUGUSTUS on the target species’ genome assembly, with the option to provide extrinsic homology and/or RNAseq evidence as hints for training (Brůna *et al.* 2021, Gabriel *et al.* 2024). Starting with BRAKER2, running the pipeline with the RNAseq data produced a better annotation than running it in the homology or RNAseq plus homology modes (Fig. 7, available as supplementary data at *Bioinformatics Advances* online); hence, its results are reported here. The BRAKER2 annotation was more complete than EVM, with 96.9% complete BUSCOs (Fig. 3A). However, it had a gene count of 17,480, which is 25.15% higher than the reference, and a larger proportion of duplicate BUSCOs (7.8%) than all except the RNAseq annotation, suggesting overprediction (Fig. 3). BRAKER3 produced a more accurate annotation, with a gene count closer to the reference and better precision scores at all feature levels (Fig. 3). Surprisingly, BRAKER3 did not outperform BRAKER2 in terms of sensitivity, with lower BUSCO completeness (86.9% versus 94.6%) and sensitivity scores (Fig. 3). Notably, while OMAnnotator (default) had better results in terms of gene content, the BRAKER3 precision scores were higher at the transcript level (44.6% versus OMAnnotator’s 38.8%), indicating BRAKER’s proficiency at modelling gene and exon borders (Fig. 3B, Table 2, available as supplementary data at *Bioinformatics Advances* online).

### 3.2 OMAnnotator re-annotates three genomes

To illustrate the practical usefulness of OMAnnotator, we re-annotated another three genomes for which source annotations were available: *S. grosvenorii* (Monk Fruit) (Xia *et al.* 2018), *P. discolor* (Pale Spear-Nosed Bat) (Jebb *et al.* 2020) and *Olea europaea* (European Olive Tree) (Cruz *et al.* 2016). All of these genomes were originally annotated using EVM with a variety of source annotation numbers and types as input (Haas *et al.* 2008). As there is no gold standard reference annotation for these non-model organisms, EVM and OMAnnotator BUSCO scores are compared with the genome assembly scores (Fig. 4).

For *S. grosvenorii*, compared to the author’s annotation set of 30,565 genes (Xia *et al.* 2018), OMAnnotator predicted 28,729 genes, closer to the mean number of 29,157 genes reported across other *Cucurbitaceae* species (Ma *et al.* 2022) (see Table 5, available as supplementary data at *Bioinformatics Advances* online, for more detailed gene content statistics). Furthermore, the OMAnnotator consensus had fewer missing BUSCO genes, with 97.0% complete BUSCOs—which was much closer to the genome assembly BUSCO completeness score (95.6%) than the EVM consensus (70.1%) (Fig. 4).

The *P. discolor* re-annotation followed the same trend: the OMAnnotator consensus had more complete BUSCOs (97.0%) than the author provided EVM consensus (96.1%) and genome assembly (95.6%) (Fig. 4). Interestingly, the BUSCO completeness score for the assembly is lower than it is for the annotations. Jebb *et al.* (2020) processed the *P. discolor* EVM annotation further to produce a final annotation with over 99.5% complete BUSCOs and 20,953 genes, which is more in line with the rest of the clade (∼20,000 genes). Based on these numbers, it is likely OMAnnotator retained false gene predictions induced by the nature of the input data (107,272 and 51,137 gene predictions in two of the four source annotations). Nevertheless, it was less prone to including false positives than EVM, with 29,062 genes in its consensus versus 62,971 genes for EVM.

For *O. europaea*, we were provided with multiple and redundant annotations from the same type of evidence (gene prediction with and without RNA/protein hints) that were used as source annotations by the authors (Cruz *et al.* 2016). Thus, we adapted our species tree input so annotations of the same type were grouped together (Fig. 10, available as supplementary data at *Bioinformatics Advances* online). The OMAnnotator annotation had 68,043 genes and 94.2% complete BUSCOs, including 24.7% duplicated. Only the authors’ already manually curated annotation was available for comparison, which was more complete with 56,349 genes and 96.7% complete BUSCOs—closer to the assembly score of 96.8% (Fig. 4). Its large number of BUSCO duplicates (23.3%) is similar to OMAnnotator’s and in line with the assembly (24.5%) (Fig. 4; Table 4, available as supplementary data at *Bioinformatics Advances* online). Cruz *et al.* (2016) suggest this large number of duplicate genes is due to extensive gene duplication in the lamiales lineage leading to the olive tree.

## 4 Discussion

We introduce OMAnnotator, an approach that repurposes OMA Standalone orthology inference software to create a consensus annotation, informed by both individual independent annotations and the gene content of other species. We validated the tool on the *D. melanogaster* genome assembly, demonstrating that the consensus annotation largely outperforms any single input annotation, as well as those made by two other established consensus annotation software run on the same inputs (Haas *et al.* 2008, Brůna *et al.* 2021, Gabriel *et al.* 2024). Finally, we tested the flexibility and robustness of OMAnnotator by using it to re-annotate three genomes that were previously annotated by EVM (Haas *et al.* 2008) using varying numbers and types of source annotations.

The quality assessment results for annotations produced by individual methods during proof of principle testing were as anticipated based on the characteristics of each approach. AUGUSTUS predicts genes using a Hidden Markov Model trained with RNAseq and homology data, making it sensitive but prone to false positives (Stanke *et al.* 2008). Here, AUGUSTUS was run with the *D. melanogaster* assembly and fly gene model parameters, i.e. a high-quality assembly and a model on which it has been extensively trained—its optimal use case. Nevertheless, it had a large number of unique predictions compared to the reference. Additionally, even with its high sensitivity, the number of missing genes was significantly higher than the OMAnnotator consensus. RNAseq data contains what is expressed in the tissues at the time of extraction, meaning genes expressed at different developmental stages can be missed, resulting in a set with more fragmented and missing BUSCO genes relative to the AUGUSTUS annotation. However, it also obtains the best results in terms of exon boundary and transcript identification. Finally, the success of homology annotation depends on the degree of relatedness between the target and the reference species and the quality of the reference proteome. In this study, we chose the *A. gambiae* proteome as our reference to mimic the situation in which the novel genome is from a species with no closely related model organism, thereby producing an annotation with lower precision and more missing genes than any other set.

The OMAnnotator consensus improves on these individual methods, filtering false gene predictions and combining true genes into a more complete consensus annotation closer to the reference than any individual method. One caveat to the BUSCO result is that OMAnnotator uses orthology data from other species to find evidence for including predicted genes in the consensus. It is therefore expected to favour predictions with highly conserved orthologs, and thus, it is expected to obtain relatively high BUSCO completeness (Manni *et al.* 2021). Nevertheless, as both the gene count and GffCompare analysis support the BUSCO result, we can be confident that the favourable BUSCO score is not solely due to this bias.

Since OMAnnotator uses homology information to select genes supported by only one of the source annotations, it is unlikely to add further support for *de novo* genes or fast evolving genes where sequence divergence is not sufficient to call homology. However, this can be compensated for by including more than one annotation source that does not use homology. Any gene supported by more than one source will be included even without support from any other species.

OMAnnotator also performed well compared to two other state-of-the-art pipelines: EVM and BRAKER (versions 2 and 3), which were chosen based on their popularity and availability of documentation (Haas *et al.* 2008, Brůna *et al.* 2021, Gabriel *et al.* 2024). Overall, OMAnnotator managed to obtain a high sensitivity compared to these other methods while keeping a high precision, particularly at the exon level. Among other methods, EVM was less prone to prediction but less sensitive than BRAKER2. This is perhaps due to its different use case: combining gene models from the user-inputted annotations and dropping spurious predictions, rather than using them as extrinsic evidence for training gene finder software. The most recent release of BRAKER3 had the TSEBRA tool integrated into its pipeline, which reduced the overprediction rate by combining gene predictions resulting from homology and RNAseq evidence (Gabriel *et al.* 2021, 2024). However, BRAKER3 also yields a less complete gene set than BRAKER2, a somewhat surprising result. Reasons are unclear, but we note that the expected use-case of BRAKER3 is homology data with a more complete database of homologues from several species, including some close relatives (Gabriel *et al.* 2024). Our test case homology dataset included only the relatively distant *A. gambiae* proteome, likely affecting its performance in our benchmark setting.

Although OMAnnotator is more accurate for gene content prediction, BRAKER3 appears more accurate in its delimitation of gene intron-exon structure, which is not modelled explicitly by OMAnnotator and can be a limitation when using it. As we demonstrated in our proof of principle testing, when good quality RNAseq data is available, OMAnnotator’s performance can be enhanced by setting RNAseq data as the priority for transcript models—this setting improved the accuracy of gene structure models beyond those of BRAKER. More importantly, since OMAnnotator can combine predictions from any GFF, it is not exclusive to any specific combination of annotation methods. We propose that OMAnnotator be used to combine annotations from methods with higher sensitivity that are specially designed to accurately predict exon-intron structures (such as BRAKER), as way to retain its ability to select most of the true genes in a consensus while not sacrificing the quality of gene model delimitation.

Any new genome annotation software designed to combine gene predictions must be flexible enough to accept multiple types of annotation sets across many species as input, yet robust enough to still filter false positives. We demonstrate that OMAnnotator possesses these qualities by re-annotating three non-model organism genomes using the authors’ source annotation sets, which were variable in number and type. It consistently predicted a number of genes similar to expectations, while maintaining BUSCO completeness scores in line with the assembly completeness.

During our benchmarking of OMAnnotator performance, we strove to acquire unprocessed annotations to allow fair comparison between EVM and OMAnnotator annotations. Several of the re-annotation candidates we identified did not have all annotations from intermediate steps publically available (or even available on request), limiting the scope of our analysis. This highlights the importance of improving reproducibility in bioinformatics pipelines, by releasing not only final data but also intermediary steps.

OMAnnotator improved upon the EVM outputs for *S. grosvenorii* and *P. discolor*, underscoring the usefulness of leveraging OMA’s orthology data. However, the final annotation for *P. discolor* and *O. europaea* had better results overall. The *P. discolor* final annotation reported by Jebb *et al.* (2020) had undergone additional rounds of PASA filtering (Haas *et al.* 2008), reducing the predicted gene count in line with expectations based on the rest of the clade and improving BUSCO results. The *O. europaea* final annotation produced by Cruz *et al.* (2016) had very few missing genes due to author curation, which OMAnnotator was unable to match despite producing a high quality gene repertoire according to BUSCO. Thus, while OMAnnotator produces highly complete and accurate first annotations by utilizing orthology evidence, downstream curation remains an important part of refining an annotation.

To conclude, by repurposing the OMA algorithm to use evolutionary data for building consensus annotations, we have created a potent new tool in the genome annotation toolbox—OMAnnotator. Its development is timely due to the continued rise of genome sequencing (Hotaling *et al.* 2021, Marks *et al.* 2021) and the widely recognized challenge of eukaryotic genome annotation (Mudge and Harrow 2016, Salzberg 2019). As OMAnnotator makes use of existing gene repertoires in evolutionary databases, we speculate its accuracy will increase as more high quality gene annotations are made available. We expect it to become a new step in annotation pipelines, where it will benefit from ongoing efforts to develop and improve gene finder methods (Scalzitti *et al.* 2020), whose annotations it can use as input datasets.

## Supplementary Material

vbag015_Supplementary_Data

## Data Availability

The data underlying this article are available in *Zenodo* at [https://doi.org/10.5281/zenodo.14252311]. This includes the OMAnnotator version and OMA Browser exported related species sets used in this study, along with all analyses and any custom scripts/notebooks.
