# Supplementary material for: OMAnnotator: a novel approach to building an annotated consensus genome sequence
Source: Bioinform Adv. 2026 Jan 22;6(1):vbag015. doi: 10.1093/bioadv/vbag015 (PMC12927413; doi:10.1093/bioadv/vbag015)
Supplement: vbag015_Supplementary_Data [file vbag015_supplementary_data.zip › 06-Feb-2026_122542_OMAnnotator_Supplementary_Material.pdf]

OMAnnotator: a novel approach to building an annotated consensus genome sequence

Supplementary Material

Proof of Principle ... 2
Figures S1-5: Species trees used for OMAnnotator performance analysis ... 2
Figure S6: OMAnnotator compute resource experiments ... 7
Figure S7: BRAKER2 results with different input combinations ... 10
Tables S1-2: Proof of principle full BUSCO results and GffCompare scores (all annotations) ... 12
Table S3: Proof of principle detailed gene content report ... 16
Re-annotation of three species ... 18
Figures S8-S10: Species trees used for OMAnnotator re-annotations ... 18
Table S4: Re-annotation of three species full BUSCO results ... 21
Table S5: Re-annotations of three species detailed gene content report ... 23
References ... 24

Proof of Principle

Figures S1-5: Species trees used for OMAnnotator performance analysis

Species trees (ordered by size; 5, 10, 15, 20 and 25 input species) used for the OMAnnotator species number runtime experiment. All trees were visualised using phylo.io (Robinson, Dylus and Dessimoz 2016) version beta.phylo.io. Manuscript results focus on the consensus produced with 25 input species.

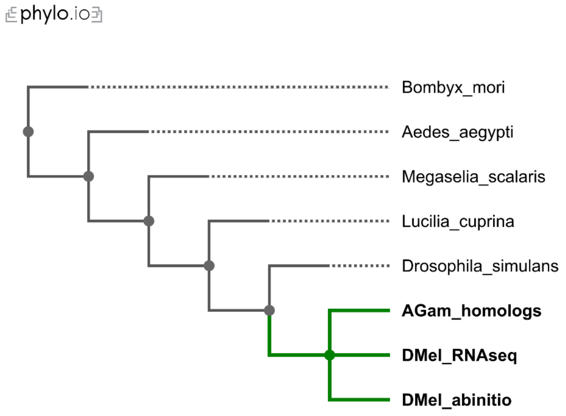

Figure S1: Proof of principle D. melanogaster OMAnnotator 5 species tree with NCBI taxonomy species names. Source Annotation (AGam_homologs, DMel_RNAseq, DMel_abinitio) branches are in green with leaf labels in bold text. The green circle is the node at which the consensus annotation is constructed.

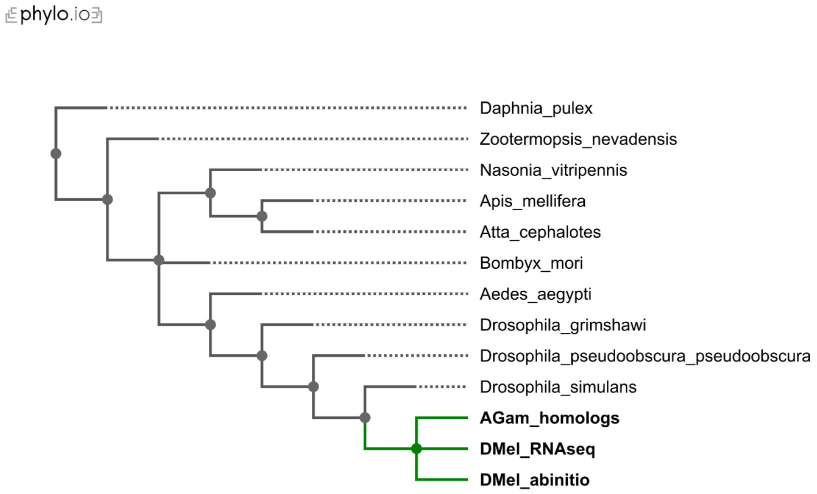

Figure S2: Proof of principle D. melanogaster OMAnnotator 10 species tree with NCBI taxonomy species names. Source Annotation (AGam_homologs, DMel_RNAseq, DMel_abinitio) branches are in green with leaf labels in bold text. The green circle is the node at which the consensus annotation is constructed.

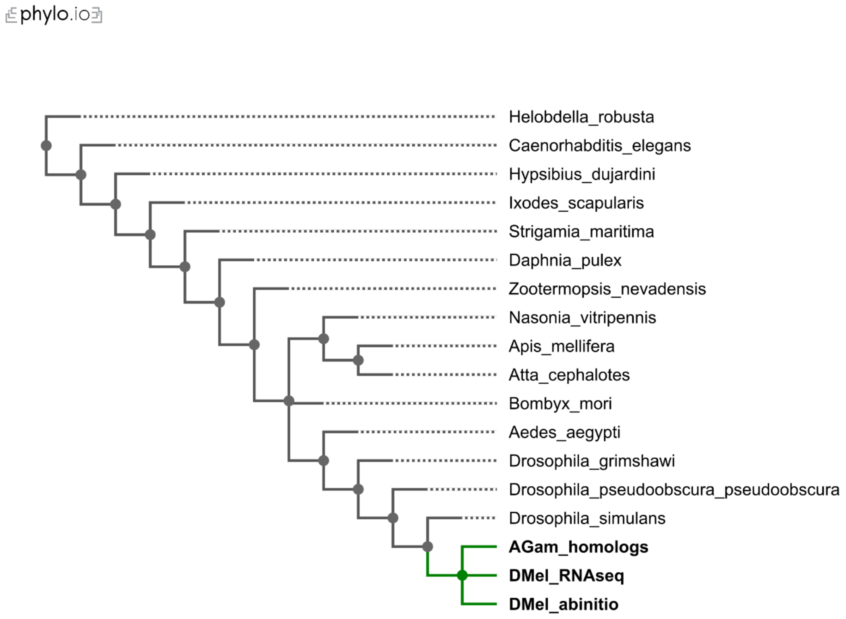

Figure S3: Proof of principle D. melanogaster OMAnnotator 15 species tree with NCBI taxonomy species names. Source Annotation (AGam_homologs, DMel_RNAseq, DMel_abinitio) branches are in green with leaf labels in bold text. The green circle is the node at which the consensus annotation is constructed.

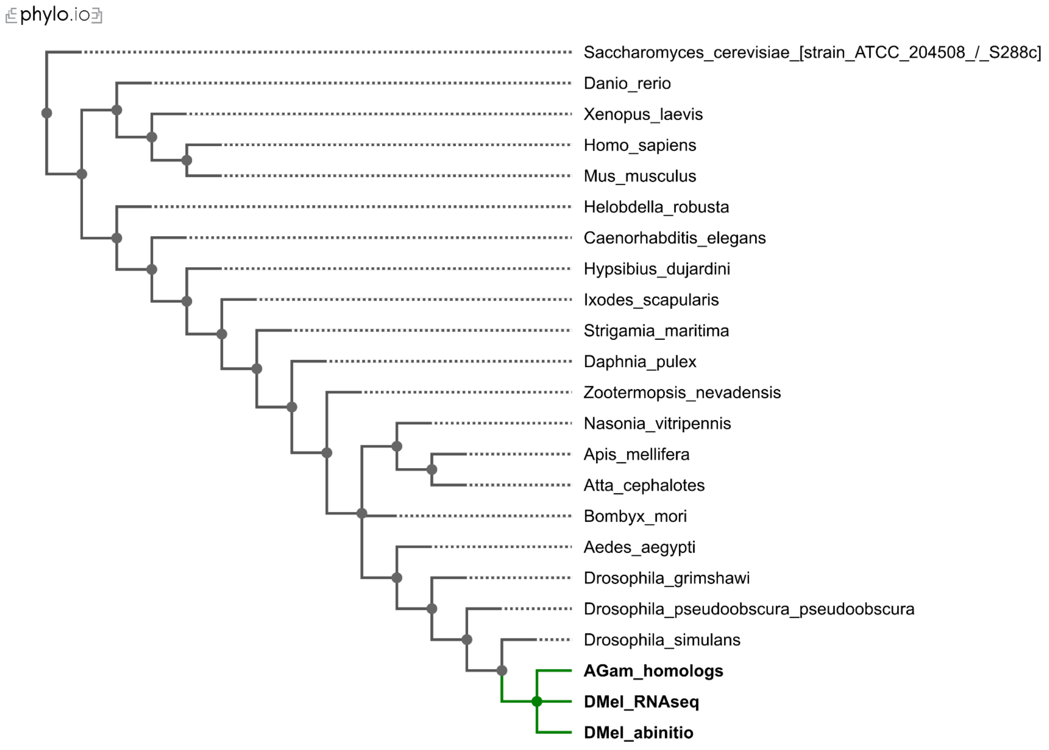

Figure S4: Proof of principle D. melanogaster OMAnnotator 20 species tree with NCBI taxonomy species names. Source Annotation (AGam_homologs, DMel_RNAseq, DMel_abinitio) branches are in green with leaf labels in bold text. The green circle is the node at which the consensus annotation is constructed.

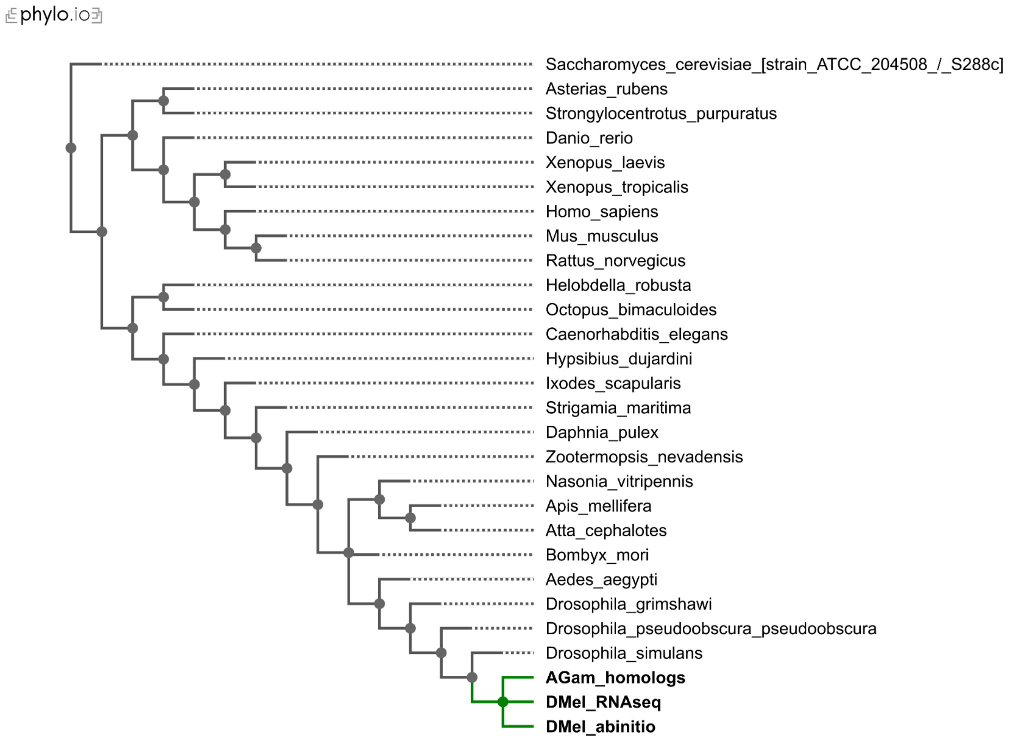

Figure S5: Proof of principle D. melanogaster OMAnnotator 25 species tree with NCBI taxonomy species names. Source Annotation (AGam_homologs, DMel_RNAseq, DMel_abinitio) branches are in green with leaf labels in bold text. The green circle is the node at which the consensus annotation is constructed.

Figure S6: OMAnnotator compute resource experiments

Results from the two runtime experiments to measure OMAnnotator's CPU hours and max RSS usage with an increasing number of 1) species and 2) source annotations (see method 2.2.5 in main manuscript).

The increase from 5-10 species yields the greatest increase in sensitivity scores (C). However, any changes are marginal. This could be due to our choice of species for the homology source annotation (*Anopheles gambiae*) being distantly related to *D. melanogaster*. Overall, using 10 species was deemed optimal, and we used 10 species for re-annotations of other species.

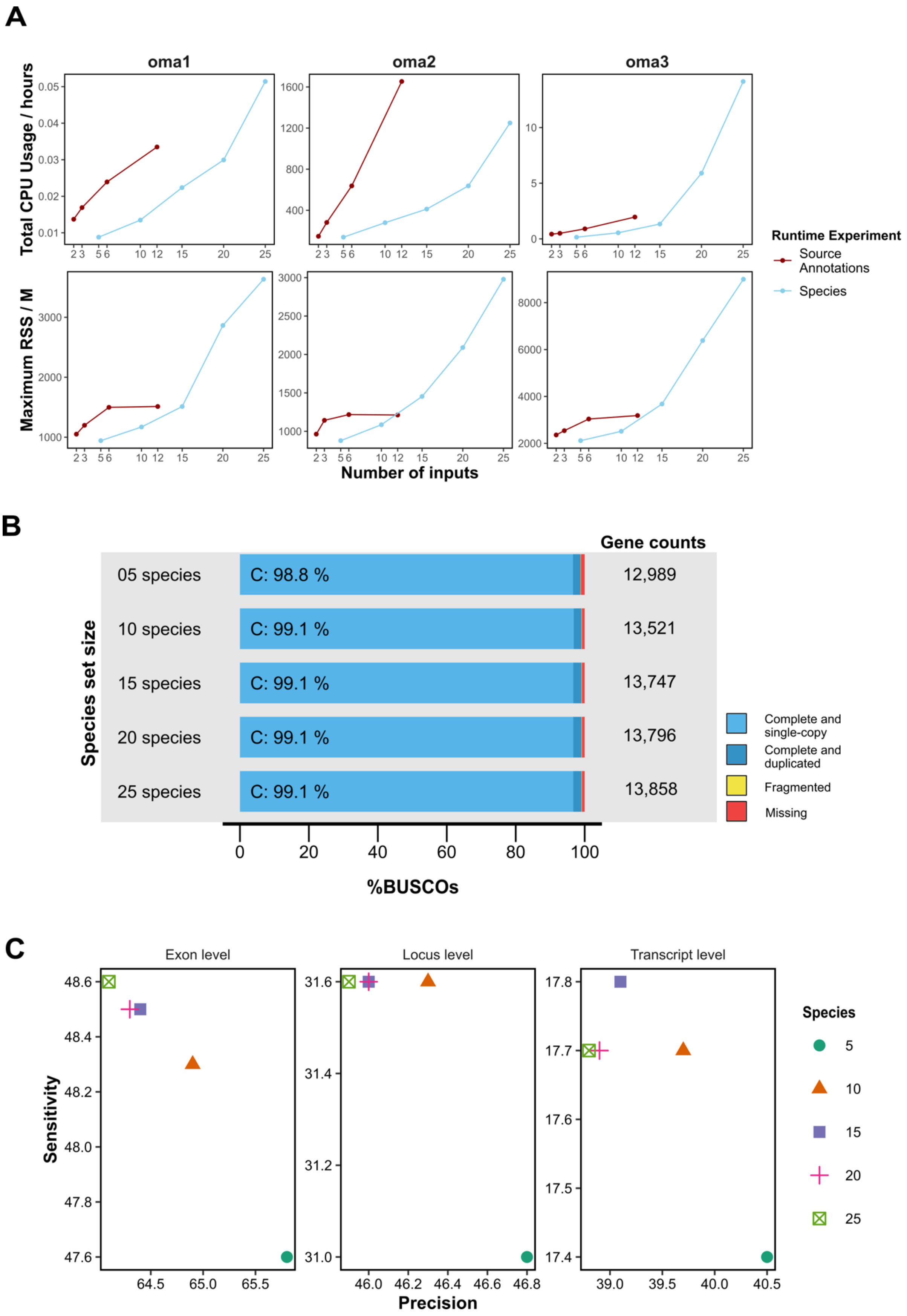

A
oma1
oma2
oma3
Total CPU Usage / hours
Maximum RSS / M
Number of inputs
Runtime Experiment
Source Annotations
Species
B
Gene counts
05 species
10 species
15 species
20 species
25 species
C: 98.8 %
C: 99.1 %
C: 99.1 %
C: 99.1 %
C: 99.1 %
12,989
13,521
13,747
13,796
13,858
Species set size
%BUSCOs
Complete and single-copy
Complete and duplicated
Fragmented
Missing
C
Exon level
Locus level
Transcript level
Sensitivity
Precision
Species
5
10
15
20
25

Figure S6: A) Compute resource usage of the OMA Standalone (orthology inference) step of OMAnnotator with increasing numbers of input species (blue) and source annotations (red). Only the OMA Standalone step is reported as it is computationally intensive enough to necessitate a HPC, whereas steps 1 and 3 of OMAnnotator can be run on a personal computer in ~1 hour. The total CPU/hours usage is shown on the top and the maximum RSS/M is shown on the bottom. OMA Standalone comprises 3 stages: database conversion (oma1), AllvsAll alignments (oma2) and Hierarchical Orthologous Group assignment (oma3). oma2 is run in parallel to reduce runtime—the total hours for 500 parallel jobs is reported here. B) BUSCO scores and gene counts C) GffCompare % Sensitivity and % Precision scores for each OMAnnotator consensus generated with different input species sets is compared with the Drosophila melanogaster reference annotation.

Figure S7: BRAKER2 results with different input combinations

Quality assessment results for three BRAKER2 annotations of the *D. melanogaster* genome assembly. BRAKER2 was run in three modes using different combinations of source annotations: RNAseq data only (RNAseq), homology data only (pep), and RNAseq and protein data (RNAseq plus pep).

The RNAseq annotation was more complete than pep, with more complete BUSCOs and higher sensitivity scores. It is also more accurate than RNAseq plus pep, with a gene count closer to the reference and fewer duplicate BUSCOs.

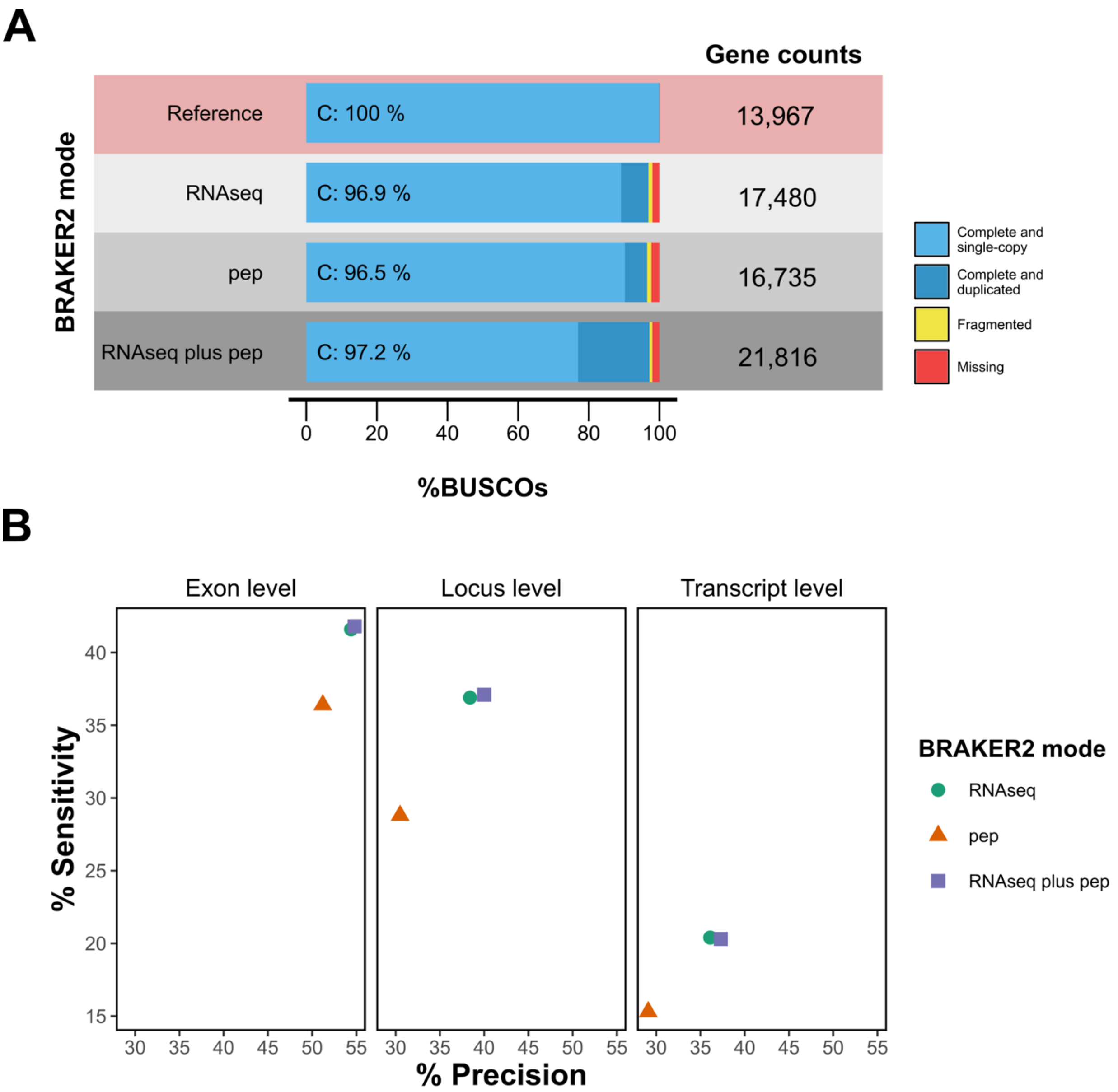

Figure S7: A) BUSCO scores and gene counts for the three BRAKER2 annotations (grey) are compared to the Drosophila melanogaster reference annotation (red). The BUSCO lineage was diptera_odb10 (3,285 BUSCOs). B) GffCompare % Sensitivity and % Precision scores for the three BRAKER2 annotations.

Tables S1-2: Proof of principle full BUSCO results and GffCompare scores (all annotations)

Annotation	BUSCO category / %				
	Single Copy	Duplicated	Fragmented	Missing	Total Complete
Reference	99.6	0.3	0.0	0.1	99.9
OMAnnotator 5 species	96.6	2.2	0.1	1.1	98.8
OMAnnotator 10 species	96.8	2.3	0.1	0.8	99.1
OMAnnotator 15 species	96.7	2.4	0.1	0.8	99.1
OMAnnotator 20 species	96.7	2.4	0.1	0.8	99.1
OMAnnotator 25 species	96.7	2.4	0.1	0.8	99.1
EVM	94.1	0.5	0.8	4.6	94.6
BRAKER2 RNAseq	89.1	7.8	1.1	2.0	96.9
BRAKER2 pep	90.2	6.3	1.2	2.3	96.5
BRAKER2 RNAseq plus pep	77.0	20.2	0.8	2.0	97.2
BRAKER3	86.4	0.5	0.7	12.4	86.9
AUGUSTUS	94.4	0.4	1.0	4.2	94.8
RNAseq	55.6	17.5	7.0	19.9	73.1

Homology	84.8	0.2	0.6	14.4	85.0

Table S1: Percentage scores for each BUSCO category for each annotation, including each OMAnnotator run with varying numbers of input species. "Total Complete" is the sum of "Single Copy" and "Duplicated" categories. Results from the OMAnnotator run with 25 species are reported in the main text as OMAnnotator (default). The BUSCO lineage was diptera_odb10 (3,285 BUSCOs).

Annotation	Base level		Exon level		Locus level		Transcript level	
	% Sn	% Prec	% Sn	% Prec	% Sn	% Prec	% Sn	% Prec
OMAnnotator 5 species	69.2	91.4	47.6	65.8	31.0	46.8	17.4	40.5
OMAnnotator 10 species	70.4	90.2	48.3	64.9	31.6	46.3	17.7	39.7
OMAnnotator 15 species	70.7	89.3	48.5	64.4	31.6	46.0	17.8	39.1
OMAnnotator 20 species	70.8	89.3	48.5	64.3	31.6	46.0	17.7	38.9
OMAnnotator 25 species	70.9	89.0	48.6	64.1	31.6	45.9	17.7	38.8
OMAnnotator (RNAseq priority)	68.6	90.3	51.8	68.8	39.3	55.6	24.3	46.5
EVM	57.7	85.2	38.9	53.6	27.5	35.3	14.6	35.3
BRAKER2 RNAseq	57.6	90.8	41.6	54.4	36.9	38.4	20.4	36.1
BRAKER2 pep	56.2	90.0	36.4	51.2	28.8	30.5	15.3	29.1

BRAKER2 RNAseq plus pep	57.6	91.0	41.8	54.8	37.1	40.0	20.3	37.3
BRAKER3	41.3	96.3	28.0	59.2	32.6	47.9	18.0	44.6
AUGUSTUS	75.2	75.2	48.1	58.1	25.4	32.3	13.5	32.3
RNAseq	47.0	95.7	38.8	73.5	33.3	56.2	21.1	50.4
Homology	33.2	98.2	18.3	41.6	8.4	18.7	4.4	18.7

Table S2: GffCompare % Sensitivity (Sn) and % Precision (Prec) scores for proof of principle annotations compared with the D. melanogaster reference annotation. OMAnnotator scores from performance testing with 5, 10, 15, 20 and 25 species in default mode are listed at the top. Results from the OMAnnotator run with 25 species are presented in the main text as OMAnnotator (default). OMAnnotator (RNAseq priority) corresponds to the annotation built by extracting the consensus from the OMAnnotator with 25 species run with the"--priority" option set to the RNAseq source annotation. This retained RNAseq gene predictions as the priority when predictions were shared by multiple source annotations.

Table S3: Proof of principle detailed gene content report

Annotation	gene counts	isoform counts	ave. isoforms/ gene	ave. transcript length/bp	prop. monoexonic
Reference	13,967	30,686	2.20	10,873	0.13
OMAnnotator 5 species	12,989	14,168	1.09	5,158	0.10
OMAnnotator 10 species	13,521	14,761	1.09	5,196	0.10
OMAnnotator 15 species	13,747	15,004	1.09	5,198	0.10
OMAnnotator 20 species	13,796	15,059	1.09	5,195	0.10
OMAnnotator 25 species	13,858	15,119	1.09	5,195	0.10
OMAnnotator (RNAseq priority)	13,858	17,622	1.27	4,572	0.11
EVM	13,355	13,355	1.00	4,799	0.20
BRAKER2 pep	16,735	17,327	1.04	2,892	0.22
BRAKER2 RNAseq	17,480	18,483	1.06	2,934	0.22
BRAKER2 RNAseq plus pep	21,816	22,417	1.03	2,865	0.16

BRAKER3	11,789	13,586	1.15	2,231	0.31
AUGUSTUS	13,530	13,530	1.00	6,785	0.12
RNAseq	10,202	14,230	1.39	32,48	0.06
Homology	7,653	7,653	1.00	3,803	0.11

Table S3: Gene content statistics for each proof of principle annotation. The number of protein-coding genes, isoforms, average isoforms per gene, average transcript length (bp) and the proportion of monoexonic genes (genes with only one exon) is reported.

Re-annotation of three species

Figures S8-S10: Species trees used for OMAnnotator re-annotations

All trees were visualised using phylo.io (Robinson, Dylus and Dessimoz 2016) version beta.phylo.io.

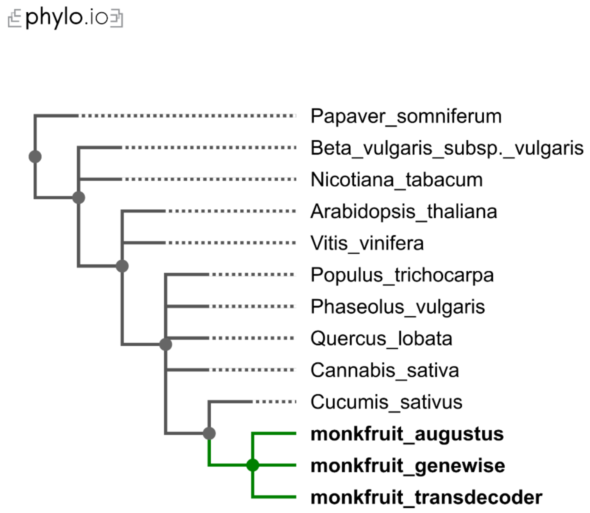

Figure S8: Siraitia grosvenorii OMAnnotator species tree. Source annotation branches are in green with leaf labels in bold text. The green circle shows the node at which the consensus annotation is constructed.

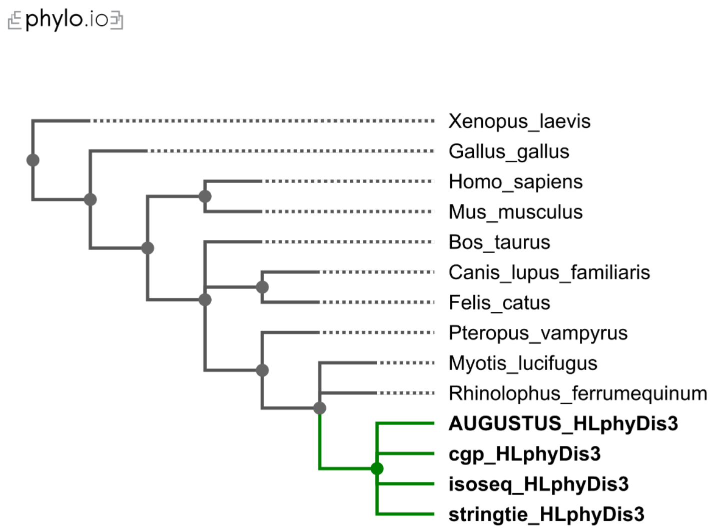

Figure S9: Phyllostomus discolor OMAnnotator species tree. Source annotation branches are in green with leaf labels in bold text. The green circle shows the node at which the consensus annotation is constructed.

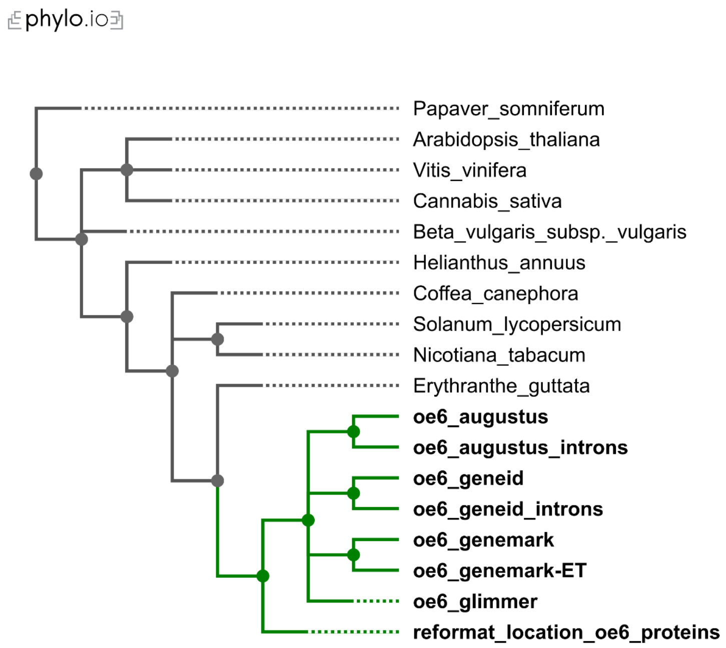

Figure S10: Olea europaea OMAnnotator species tree. Source annotation branches are in green with leaf labels in bold text. The green circle shows the node at which the consensus annotation is constructed.

Table S4: Re-annotation of three species full BUSCO results

Species	Format	BUSCO category / %				
		S	D	F	M	Total Complete
Siraitia grosvenorii	Assembly	59.0	36.6	1.2	3.2	95.6
	Author Annotation	48.7	21.4	5.4	24.5	70.1
	OMAnnotator	72.7	12.5	6.5	8.3	85.2
Phyllostomus discolor	Assembly	94.8	0.8	0.6	3.8	95.6
	Author Annotation	44.1	52.0	0.3	3.6	96.1
	OMAnnotator	95.5	1.5	0.7	2.3	97.0
Olea europaea	Assembly	72.3	24.5	1.7	1.5	96.8
	Author Annotation	73.4	23.3	1.2	2.1	96.7
	OMAnnotator	69.5	24.7	2.9	2.9	94.2

Table S4: Complete single copy (S), complete duplicated (D), fragmented (F), missing (M) and Total Complete (S+D) BUSCO category percentages for Siraitia grosvenorii, Phyllostomus discolor and Olea europaea reannotations. Author and OMAnnotator annotations are compared to the Assembly BUSCO scores. BUSCO lineages were eudicots_odb10 (2,326 BUSCOs) for

Siraitia grosvenorii and Olea europaea, and laurasiatheria_odb10 (12,234 BUSCOs) for Phyllostomus discolor.

Table S5: Re-annotations of three species detailed gene content report

Species	Annotation	gene counts	isoform counts	ave. isoforms/ gene	ave. transcript length/bp	prop. monoexonic
Siraitia grosvenorii	Author consensus	30,565	30,565	1.00	4,525	0.21
	OMAnnotator	28,729	60,017	2.09	9,307	0.17
Phyllostomus discolor	Author consensus	62,971	62,971	1.00	52,495	0.08
	OMAnnotator	29,062	33,321	1.15	33,836	0.18
Olea europaea	Author consensus	56,349	89,982	1.60	4,848	0.21
	OMAnnotator	68,043	68,043	1.00	7,889	0.06

Table S5: Gene content statistics for the three re-annotations. The number of protein-coding genes, isoforms, average isoforms per gene, average transcript length (bp) and the proportion of monexonic genes (genes with only one exon) is reported for the Authors' annotations and the OMAnnotator re-annotations.

References

Robinson O, Dylus D, Dessimoz C. Phylo.io : Interactive Viewing and Comparison of Large Phylogenetic Trees on the Web. *Mol Biol Evol* 2016;**33**:2163–6.
