# Supplementary material for: OMAnnotator: a novel approach to building an annotated consensus genome sequence
Source: Bioinform Adv. 2026 Jan 22;6(1):vbag015. doi: 10.1093/bioadv/vbag015 (PMC12927413; doi:10.1093/bioadv/vbag015)
Supplement: vbag015_Supplementary_Data [file vbag015_supplementary_data.zip › Copy_of_OMAnnotator_Supplementary_Material_v2_unlinked.docx]

OMAnnotator: a novel approach to building an annotated consensus genome sequence

Supplementary Material

#

[**Proof of Principle 2**](#_bky3e4dza8e8)

[Figures S1-5: Species trees used for OMAnnotator runtime analysis 2](#_bdxnnv6yfaen)

[Figure S6: OMAnnotator compute resource experiments 7](#_2ddy10n7y4xb)

[Figure S7: BRAKER2 results with different input combinations 10](#_936spq1lm2hs)

[Tables S1-2: Full BUSCO and GffCompare scores, all annotations 12](#_66xtu0e5d4zn)

[Table S3: Proof of principle detailed gene content report 15](#_6oxwk3nzanut)

[**Re-annotation of three species 17**](#_xaeekq5266jn)

[Figures S8-S10: Species trees used for OMAnnotator re-annotations 17](#_mc2rdrduus1u)

[Table S4: Re-annotation of three species BUSCO results 20](#_yhm616ea0gfr)

[Table S5: Re-annotations of three species detailed gene content report 21](#_7if6w314x2lk)

[**References 22**](#_t9772cqgvujj)

##

# Proof of Principle

##

## Figures S1-5: Species trees used for OMAnnotator runtime analysis

Species trees (ordered by size; 5, 10, 15, 20 and 25 input species) used for the OMAnnotator species number runtime experiment. All trees were visualised using phylo.io [(Robinson, Dylus and Dessimoz 2016)](https://www.zotero.org/google-docs/?cISAhV) version [beta.phylo.io](http://beta.phylo.io). Manuscript results focus on the consensus produced with 25 input species.

##
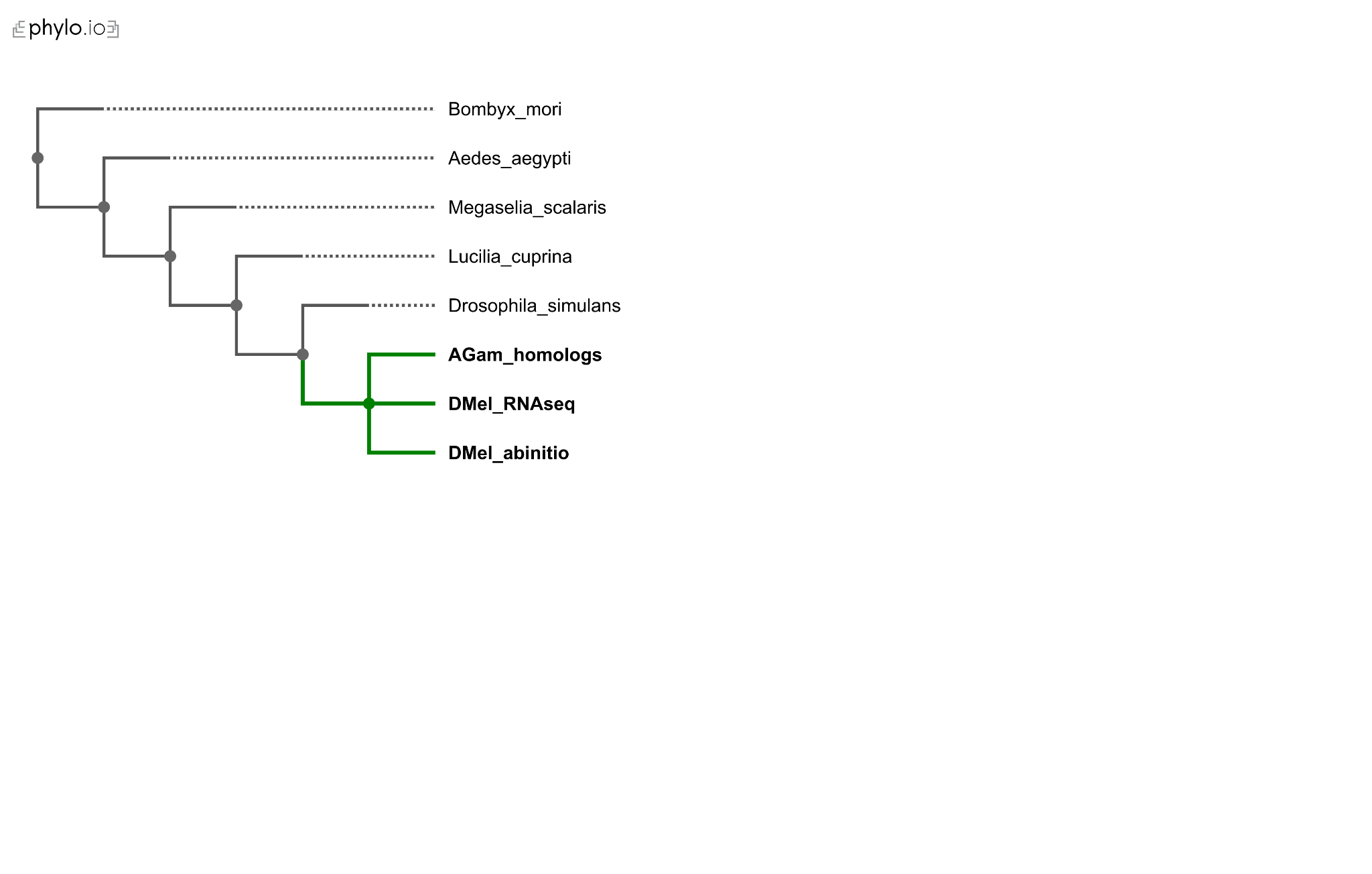


*Figure S1: Proof of principle D. melanogaster OMAnnotator 5 species tree with NCBI taxonomy species names. Source Annotation (AGam_homologs, DMel_RNAseq, DMel_abinitio) branches are in green with leaf labels in bold text. The green circle is the node at which the consensus annotation is constructed.*


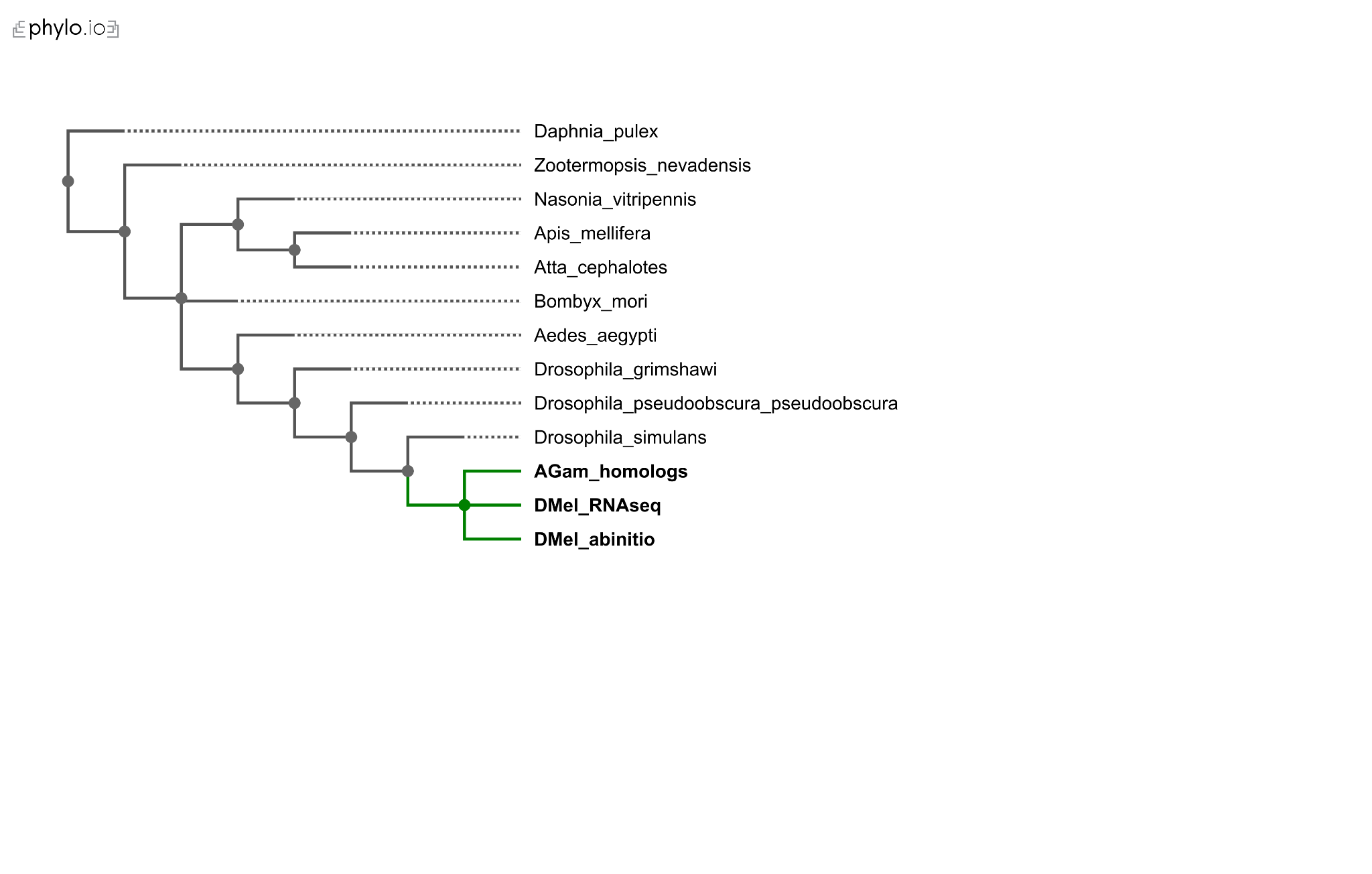


*Figure S2: Proof of principle D. melanogaster OMAnnotator 10 species tree with NCBI taxonomy species names. Source Annotation (AGam_homologs, DMel_RNAseq, DMel_abinitio) branches are in green with leaf labels in bold text. The green circle is the node at which the consensus annotation is constructed.*


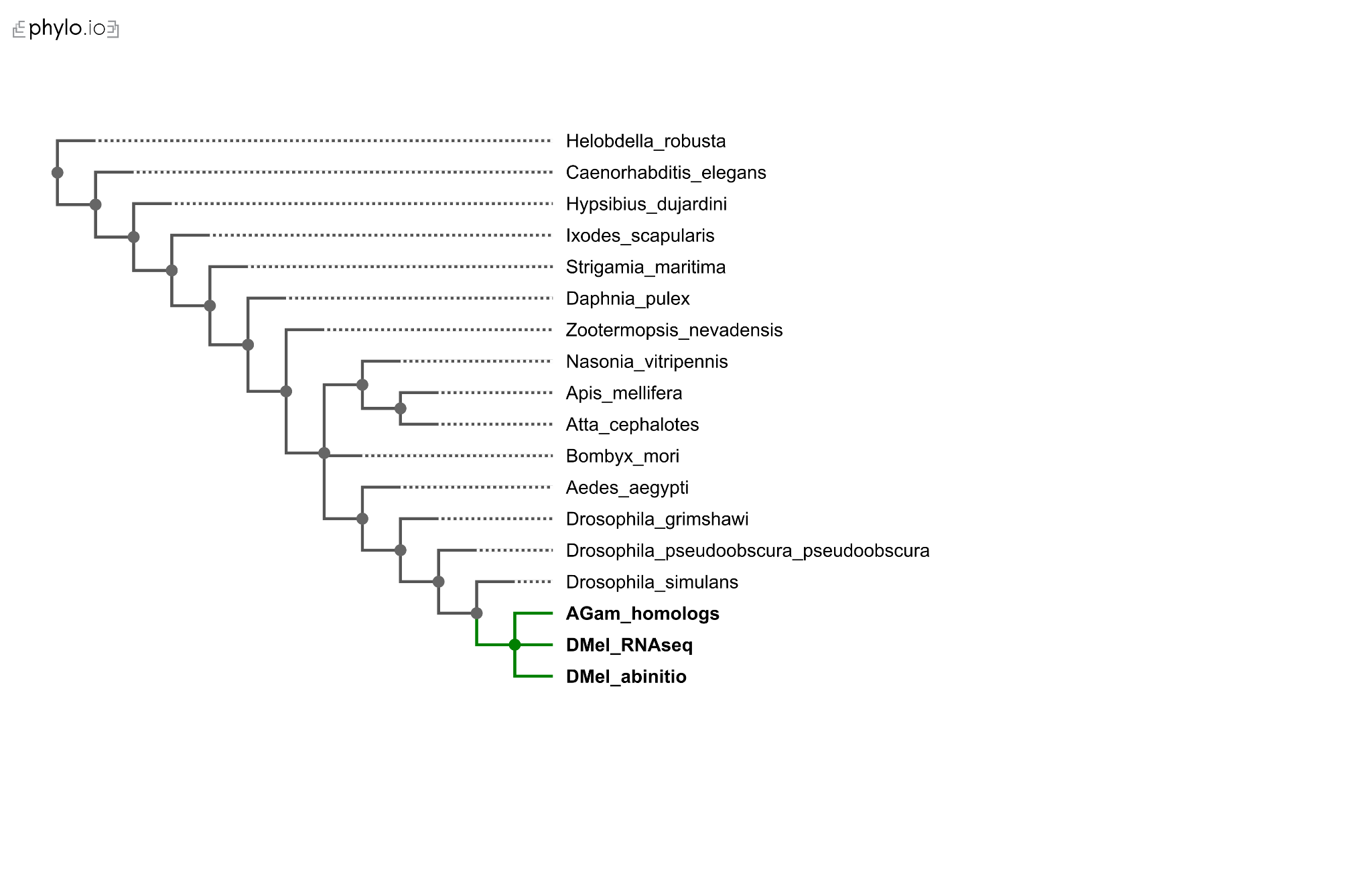


*Figure S3: Proof of principle D. melanogaster OMAnnotator 15 species tree with NCBI taxonomy species names. Source Annotation (AGam_homologs, DMel_RNAseq, DMel_abinitio) branches are in green with leaf labels in bold text. The green circle is the node at which the consensus annotation is constructed.*


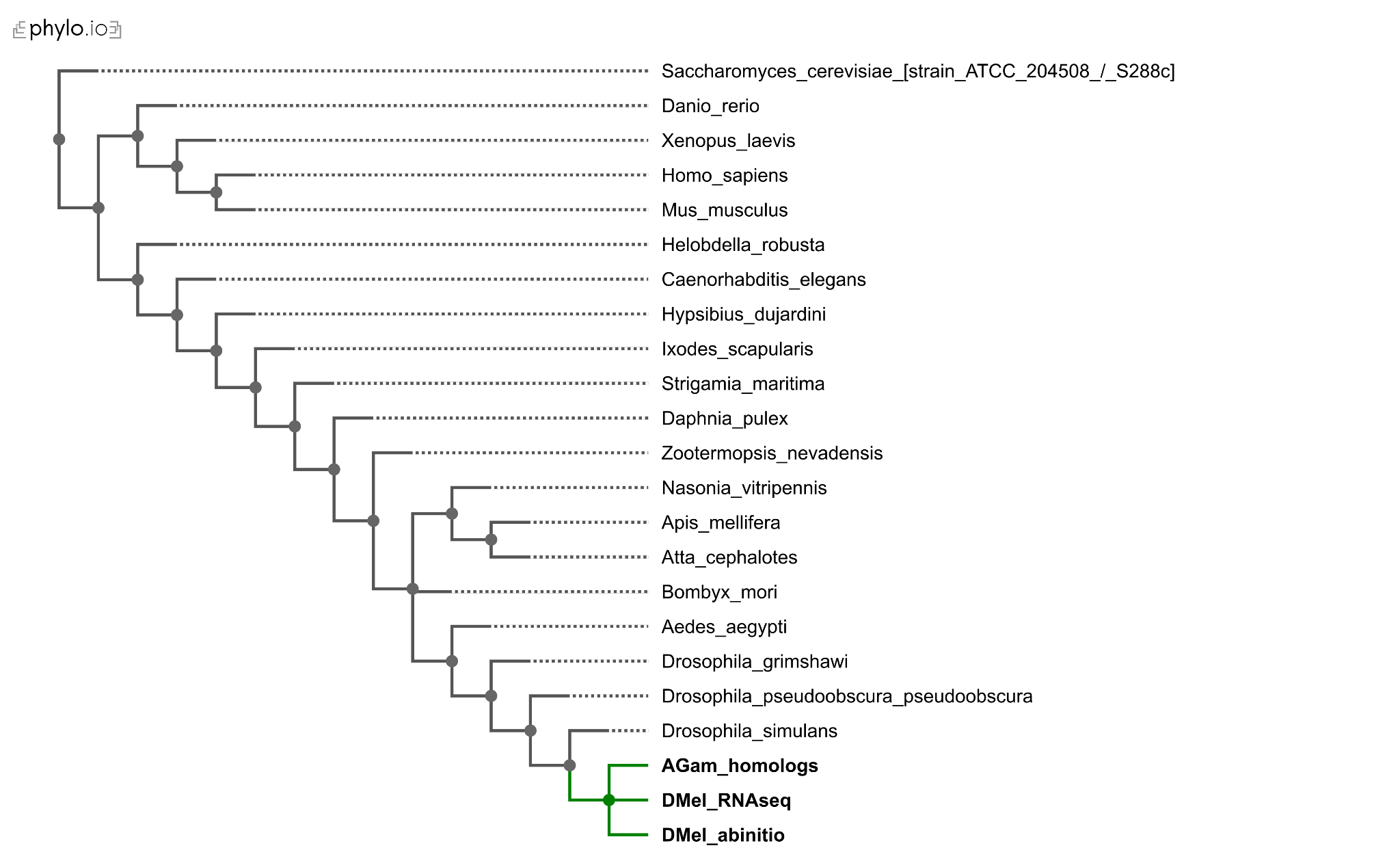


*Figure S4: Proof of principle D. melanogaster OMAnnotator 20 species tree with NCBI taxonomy species names. Source Annotation (AGam_homologs, DMel_RNAseq, DMel_abinitio) branches are in green with leaf labels in bold text. The green circle is the node at which the consensus annotation is constructed.*


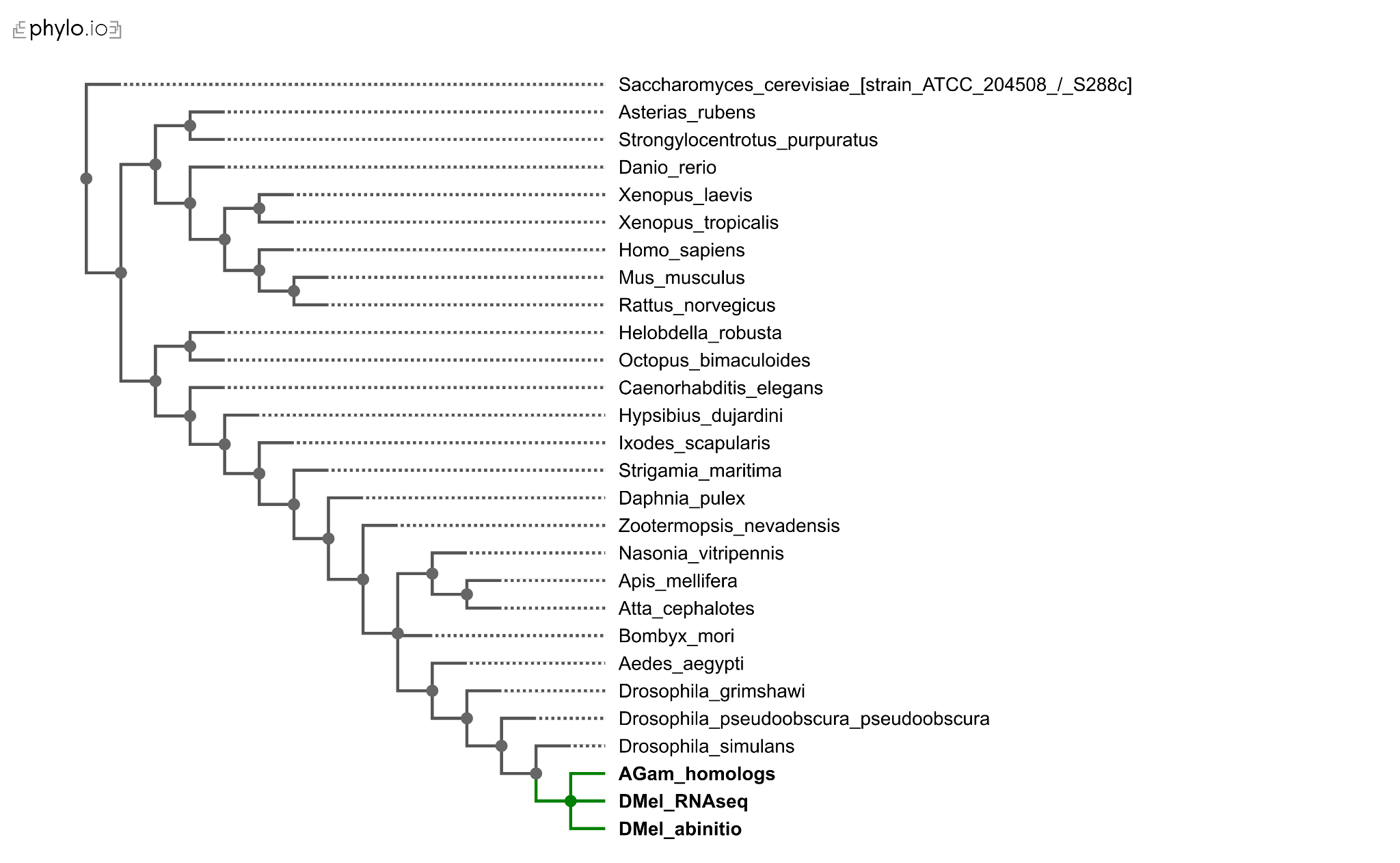


*Figure S5: Proof of principle D. melanogaster OMAnnotator 25 species tree with NCBI taxonomy species names. Source Annotation (AGam_homologs, DMel_RNAseq, DMel_abinitio) branches are in green with leaf labels in bold text. The green circle is the node at which the consensus annotation is constructed.*

## Figure S6: OMAnnotator compute resource experiments

Results from the two runtime experiments to measure OMAnnotator’s CPU hours and max RSS usage with an increasing number of 1) species and 2) source annotations (see method 2.2.5 in main manuscript).

The increase from 5-10 species yields the greatest increase in sensitivity scores (C). However, any changes are marginal. This could be due to our choice of species for the homology source annotation (*Anopheles gambiae*) being distantly related to *D. melanogaster*. Overall, using 10 species was deemed optimal, and we used 10 species for re-annotations of other species.

*
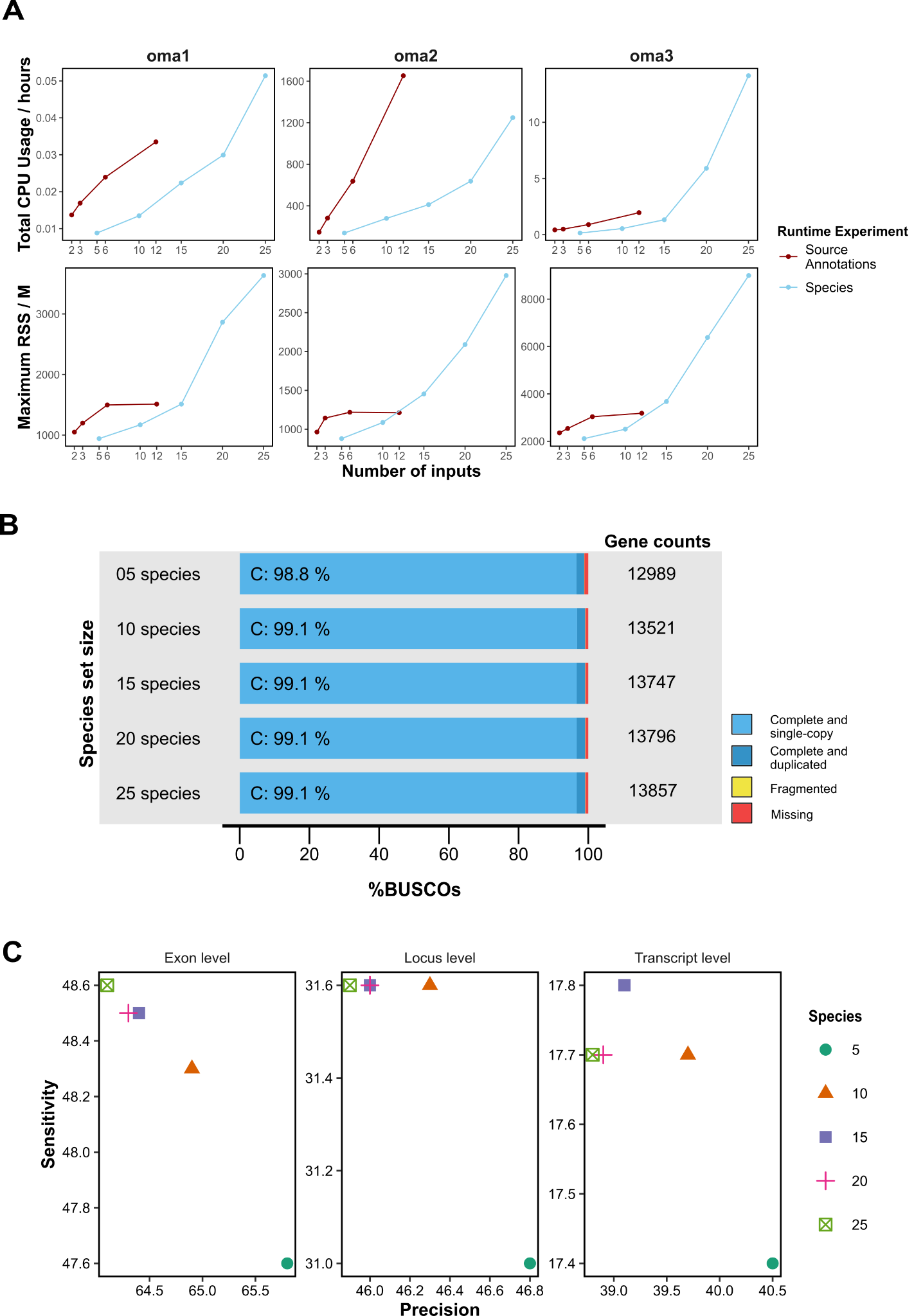
*

*Figure S6: A) Compute resource usage of the OMA Standalone (orthology inference) step of OMAnnotator with increasing numbers of input species (blue) and source annotations (red). Only the OMA Standalone step is reported as it is computationally intensive enough to necessitate a HPC, whereas steps 1 and 3 of OMAnnotator can be run on a personal computer in ~1 hour. The total CPU/hours usage is shown on the top and the maximum RSS/M is shown on the bottom. OMA Standalone comprises 3 stages: database conversion (oma1), AllvsAll alignments (oma2) and Hierarchical Orthologous Group assignment (oma3). oma2 is run in parallel to reduce runtime—the total hours for 500 parallel jobs is reported here. B) BUSCO scores and gene counts C) GffCompare Sensitivity and Precision statistics for each OMAnnotator consensus generated with different input species sets is compared with the Drosophila melanogaster reference annotation.*

## Figure S7: BRAKER2 results with different input combinations

##

Quality assessment results for three BRAKER2 annotations of the *D. melanogaster* genome assembly. BRAKER2 was run in three modes using different combinations of source annotations: RNAseq data only (RNAseq), homology data only (pep), and RNAseq and protein data (RNAseq plus pep).

The RNAseq annotation was more complete than pep, with more complete BUSCOs and higher sensitivity scores. It is also more accurate than RNAseq plus pep, with a gene count closer to the reference and fewer duplicate BUSCOs.

**
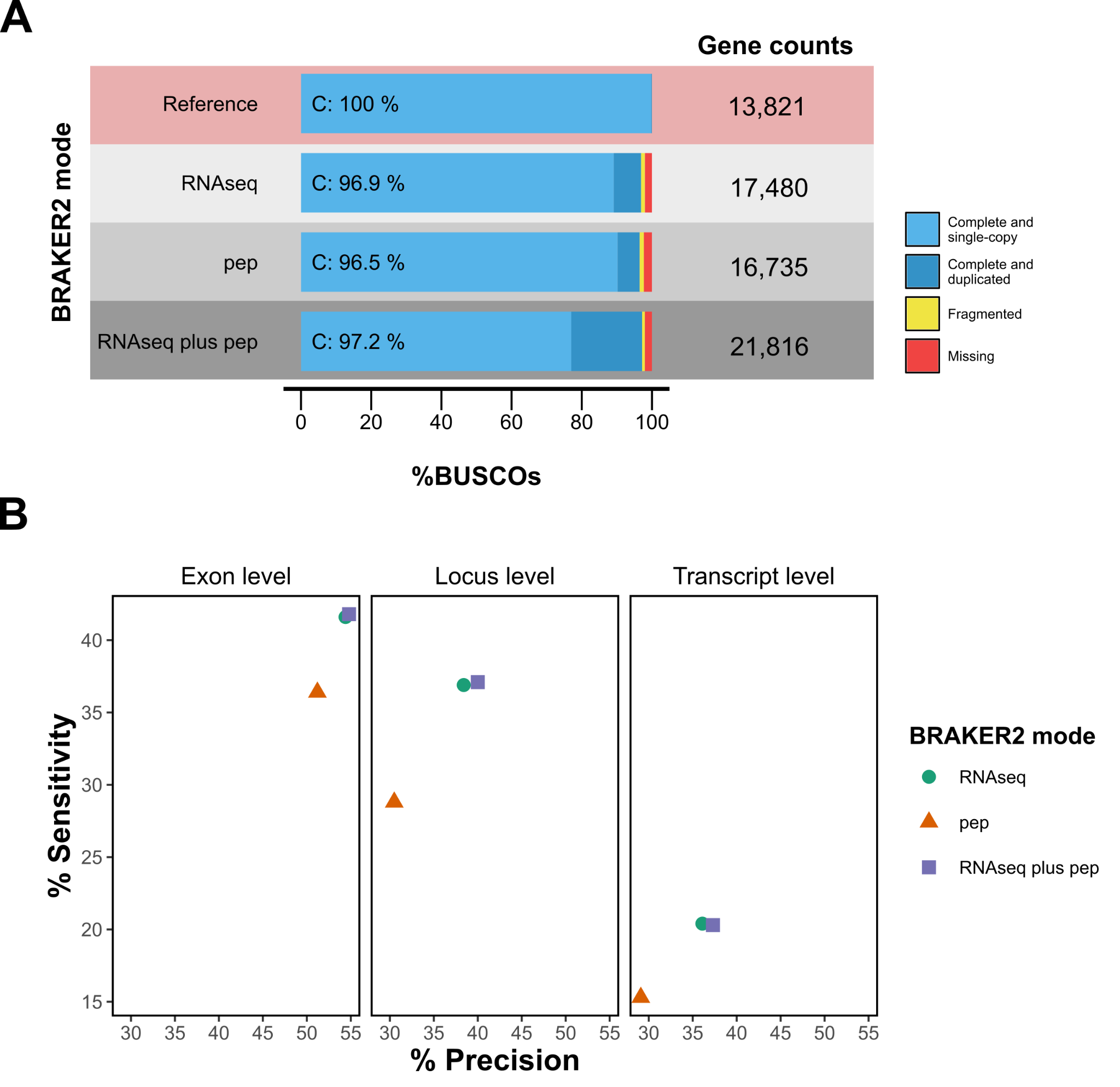
**

*Figure S7: A) BUSCO scores and gene counts for the three BRAKER2 annotations (grey) are compared to the Drosophila melanogaster reference annotation (red). The BUSCO lineage was diptera_odb10 (3,285 BUSCOs). B) GffCompare % Sensitivity (Sn) and % Precision (Prec) scores at the Exon, Transcript and Locus level for the three BRAKER2 annotations.*

## Tables S1-2: Full BUSCO and GffCompare scores, all annotations

| **Annotation** | **BUSCO category / %** | | | | |
| --- | --- | --- | --- | --- | --- |
|  | **Single Copy** | **Duplicated** | **Fragmented** | **Missing** | **Total Complete** |
| Reference | 99.8 | 0.2 | 0.0 | 0.0 | 100.0 |
| OMAnnotator  5 species | 96.6 | 2.2 | 0.1 | 1.1 | 98.8 |
| OMAnnotator  10 species | 96.8 | 2.3 | 0.1 | 0.8 | 99.1 |
| OMAnnotator  15 species | 96.7 | 2.4 | 0.1 | 0.8 | 99.1 |
| OMAnnotator  20 species | 96.7 | 2.4 | 0.1 | 0.8 | 99.1 |
| OMAnnotator  25 species | 96.7 | 2.4 | 0.1 | 0.8 | 99.1 |
| EVM | 94.1 | 0.5 | 0.8 | 4.6 | 94.6 |
| BRAKER2 RNAseq | 89.1 | 7.8 | 1.1 | 2.0 | 96.9 |
| BRAKER2 pep | 90.2 | 6.3 | 1.2 | 2.3 | 96.5 |
| BRAKER2 RNAseq plus pep | 77.0 | 20.2 | 0.8 | 2.0 | 97.2 |
| BRAKER3 | 86.4 | 0.5 | 0.7 | 12.4 | 86.9 |
| AUGUSTUS | 94.4 | 0.4 | 1.0 | 4.2 | 94.8 |
| RNAseq | 55.6 | 17.5 | 7.0 | 19.9 | 73.1 |
| Homology | 84.8 | 0.2 | 0.6 | 14.4 | 85.0 |

*Table S1: Percentage scores for each BUSCO category for each annotation, including each OMAnnotator run with varying numbers of input species.“Total Complete” is the sum of “Single Copy” and “Duplicated” categories. Results from the OMAnnotator run with 25 species are reported in the main text as OMAnnotator (default). The BUSCO lineage was diptera_odb10 (3285 BUSCOs).*

| **Annotation** | **Base level** | | **Exon level** | | **Locus level** | | **Transcript level** | |
| --- | --- | --- | --- | --- | --- | --- | --- | --- |
|  | **% Sn** | **% Prec** | **% Sn** | **% Prec** | **% Sn** | **% Prec** | **% Sn** | **% Prec** |
| OMAnnotator  5 species | 69.2 | 91.4 | 47.6 | 65.8 | 31.0 | 46.8 | 17.4 | 40.5 |
| OMAnnotator  10 species | 70.4 | 90.2 | 48.3 | 64.9 | 31.6 | 46.3 | 17.7 | 39.7 |
| OMAnnotator  15 species | 70.7 | 89.3 | 48.5 | 64.4 | 31.6 | 46.0 | 17.8 | 39.1 |
| OMAnnotator  20 species | 70.8 | 89.3 | 48.5 | 64.3 | 31.6 | 46.0 | 17.7 | 38.9 |
| OMAnnotator  25 species | 70.9 | 89.0 | 48.6 | 64.1 | 31.6 | 45.9 | 17.7 | 38.8 |
| OMAnnotator (RNAseq priority) | 68.6 | 90.3 | 51.8 | 68.8 | 39.3 | 55.6 | 24.3 | 46.5 |
| EVM | 57.7 | 85.2 | 38.9 | 53.6 | 27.5 | 35.3 | 14.6 | 35.3 |
| BRAKER2  RNAseq | 57.6 | 90.8 | 41.6 | 54.4 | 36.9 | 38.4 | 20.4 | 36.1 |
| BRAKER2  pep | 56.2 | 90.0 | 36.4 | 51.2 | 28.8 | 30.5 | 15.3 | 29.1 |
| BRAKER2  RNAseq plus pep | 57.6 | 91.0 | 41.8 | 54.8 | 37.1 | 40.0 | 20.3 | 37.3 |
| BRAKER3 | 41.3 | 96.3 | 28.0 | 59.2 | 32.6 | 47.9 | 18.0 | 44.6 |
| AUGUSTUS | 75.2 | 75.2 | 48.1 | 58.1 | 25.4 | 32.3 | 13.5 | 32.3 |
| RNAseq | 47.0 | 95.7 | 38.8 | 73.5 | 33.3 | 56.2 | 21.1 | 50.4 |
| Homology | 33.2 | 98.2 | 18.3 | 41.6 | 8.4 | 18.7 | 4.4 | 18.7 |

*Table S2: GffCompare scores for proof of principle annotations compared with the D. melanogaster reference annotation. Scores given as percentages. OMAnnotator scores from performance testing with 5,10,15,20 and 25 species in default mode are listed at the top. Results from the OMAnnotator run with 25 species are presented in the main text as OMAnnotator (default). OMAnnotator (RNAseq priority) corresponds to the annotation built by extracting the consensus from the OMAnnotator with 25 species run with the“-priority” option set to the RNAseq source annotation. This retained RNAseq gene predictions as priority when predictions were shared by multiple source annotations.*

#

## Table S3: Proof of principle detailed gene content report

| **Annotation** | **gene counts** | **isoform counts** | **ave. isoforms/gene** | **ave. transcript length/bp** | **prop.**  **monoexonic** |
| --- | --- | --- | --- | --- | --- |
| Reference | 13,967 | 30,686 | 2.2 | 10,873 | 0.29 |
| OMAnnotator  5 species | 12,989 | 14,168 | 1.09 | 5,158 | 0.09 |
| OMAnnotator  10 species | 13,521 | 14,761 | 1.09 | 5,196 | 0.09 |
| OMAnnotator  15 species | 13,747 | 15,004 | 1.09 | 5,198 | 0.09 |
| OMAnnotator  20 species | 13,796 | 15,059 | 1.09 | 5,195 | 0.09 |
| OMAnnotator  25 species | 13,858 | 15,119 | 1.09 | 5,195 | 0.09 |
| OMAnnotator (RNAseq priority) | 13,858 | 17,622 | 1.27 | 4,572 | 0.1 |
| EVM | 13,355 | 13,355 | 1 | 4,799 | 0.2 |
| BRAKER2  pep | 16,735 | 17,327 | 1.04 | 2,892 | 0.22 |
| BRAKER2  RNAseq | 17,480 | 18,483 | 1.06 | 2,934 | 0.22 |
| BRAKER2  RNAseq plus pep | 21,816 | 22,417 | 1.03 | 2,865 | 0.15 |
| BRAKER3 | 11,789 | 13,586 | 1.15 | 2,231 | 0.31 |
| AUGUSTUS | 13,530 | 13,530 | 1 | 6,785 | 0.12 |
| RNAseq | 10,202 | 14,230 | 1.39 | 3,248 | 0.06 |
| Homology | 7,653 | 7,653 | 1 | 3,803 | 0 |

##

*Table S3: Gene content statistics for each proof of principle annotation. The number of genes, isoforms, average isoforms per gene, average transcript length (bp) and the proportion of monexonic genes (genes with only one exon) is reported.*

# Re-annotation of three species

## Figures S8-S10: Species trees used for OMAnnotator re-annotations

All trees were visualised using phylo.io [(Robinson, Dylus and Dessimoz 2016)](https://www.zotero.org/google-docs/?WgAMGu) version [beta.phylo.io](http://beta.phylo.io).


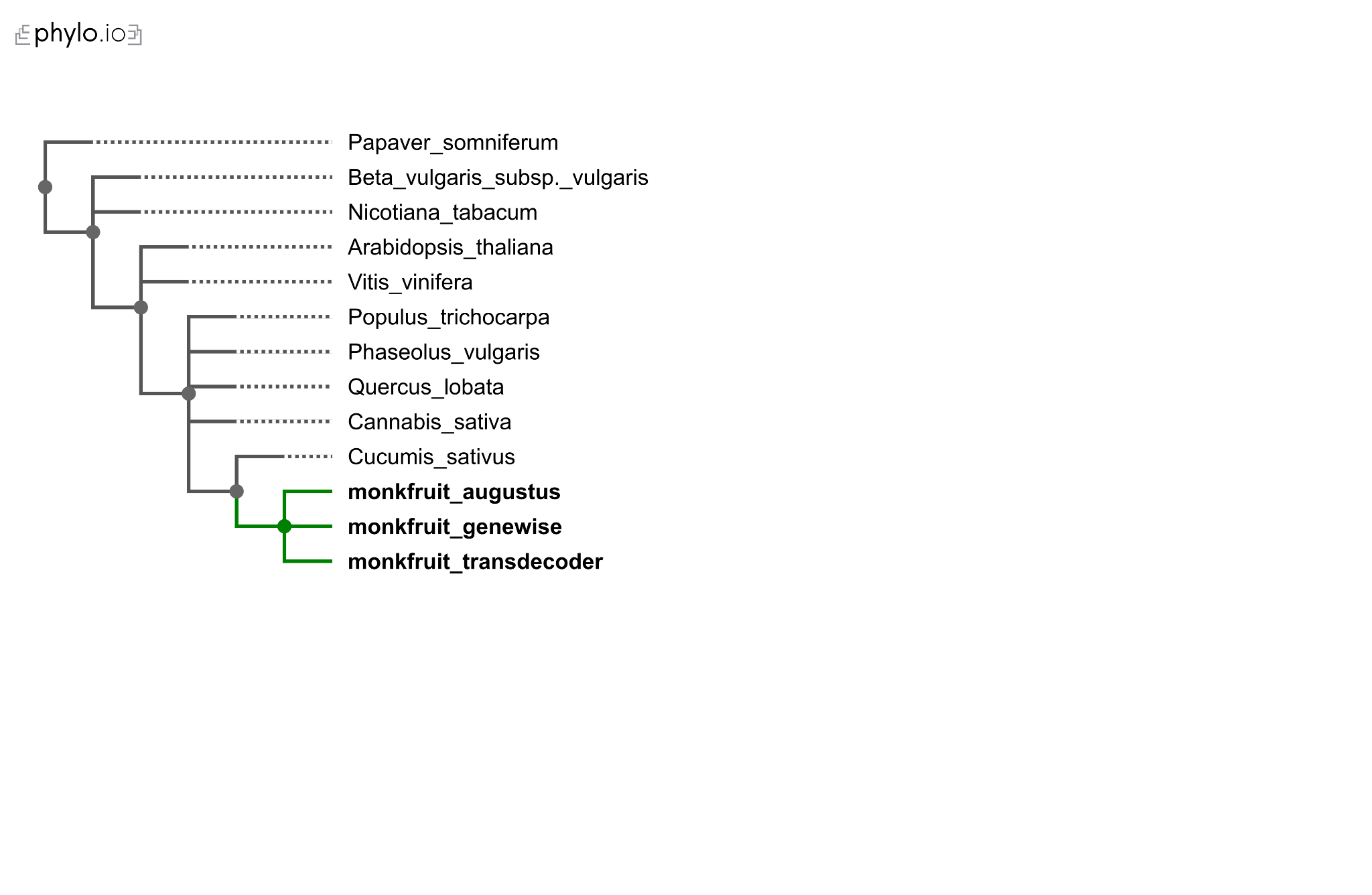


*Figure S8: Siraitia grosvenorii OMAnnotator species tree. Source annotations are highlighted by the dashed box and black circles. The green circle shows the node at which the consensus annotation is constructed.*


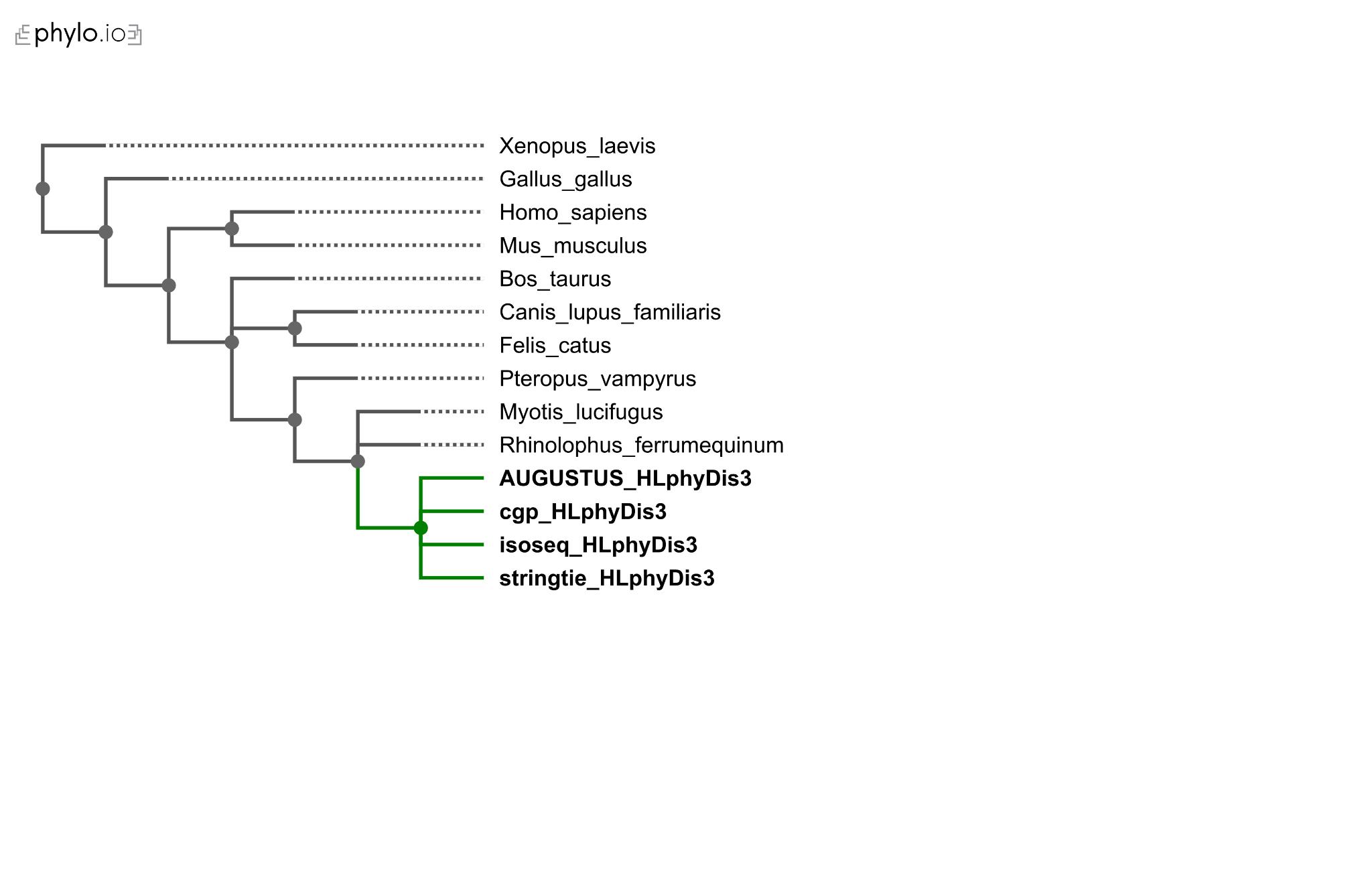


*Figure S9: Phyllostomus discolor OMAnnotator species tree. Source annotations are highlighted by the dashed box and black circles. The green circle shows the node at which the consensus annotation is constructed.*


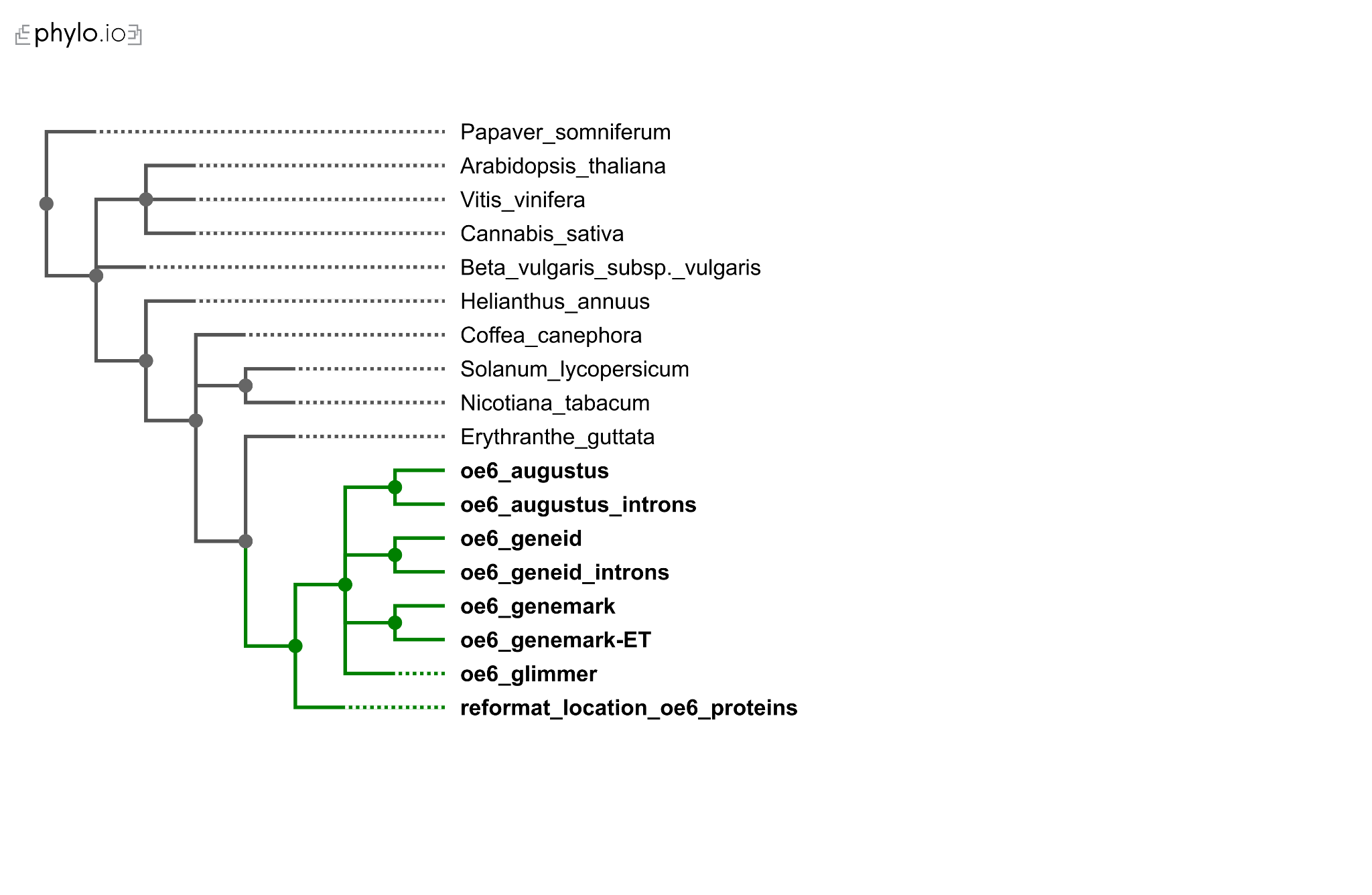


*Figure S10: Olea europaea OMAnnotator species tree. Source annotations are highlighted by the dashed box and black circles. The green circle shows the node at which the consensus annotation is constructed.*

## Table S4: Re-annotation of three species BUSCO results

##

| **Species** | **Format** | **BUSCO category / %** | | | | |
| --- | --- | --- | --- | --- | --- | --- |
|  |  | **S** | **D** | **F** | **M** | **Total Complete** |
| ***Siraitia grosvenorii*** | Assembly | 59.0 | 36.6 | 1.2 | 3.2 | 95.6 |
|  | Author Annotation | 48.7 | 21.4 | 5.4 | 24.5 | 70.1 |
|  | OMAnnotator | 72.7 | 12.5 | 6.5 | 8.3 | 85.2 |
| ***Phyllostomus discolor*** | Assembly | 94.8 | 0.8 | 0.6 | 3.8 | 95.6 |
|  | Author Annotation | 44.1 | 52.0 | 0.3 | 3.6 | 96.1 |
|  | OMAnnotator | 95.5 | 1.5 | 0.7 | 2.3 | 97 |
| ***Olea***  ***europaea*** | Assembly | 72.3 | 24.5 | 1.7 | 1.5 | 96.8 |
|  | Author Annotation | 73.4 | 23.3 | 1.2 | 2.1 | 96.7 |
|  | OMAnnotator | 69.5 | 24.7 | 2.9 | 2.9 | 94.2 |

*Table S4: Complete single copy (S), complete duplicated (D), fragmented, missing and Total Complete BUSCO category percentages for Siraitia grosvenorii, Phyllostomus discolor,, and Olea europaea reannotations. Author and OMAnnotator annotations are compared to the Assembly BUSCO scores. BUSCO lineages were eudicots_odb10 (2,326 BUSCOs) for Siraitia grosvenorii and Olea europaea, and laurasiatheria_odb10 (12,234 BUSCOs) for Phyllostomus discolor.*

## Table S5: Re-annotations of three species detailed gene content report

##

| **Species** | **Annotation** | **gene counts** | **isoform counts** | **ave. isoforms/gene** | **ave. transcript length/bp** | **prop.**  **monoexonic** |
| --- | --- | --- | --- | --- | --- | --- |
| ***Siraitia grosvenorii*** | Author  consensus | 30,565 | 30,565 | 1 | 4,525 | 0.21 |
|  | OMAnnotator | 23,027 | 54,315 | 2.36 | 9,537 | 0.02 |
| ***Phyllostomus discolor*** | Author  consensus | 62,971 | 62,971 | 1 | 52,495 | 0.08 |
|  | OMAnnotator | 25,305 | 33,321 | 1.32 | 33,836 | 0.16 |
| ***Olea***  ***europaea*** | Author  consensus | 56,349 | 89,982 | 1.6 | 4,848 | 0.21 |
|  | OMAnnotator | 66,319 | 67,481 | 1.02 | 7,892 | 0.06 |

*Table S5: Gene content statistics for the three re-annotations. The number of genes, isoforms, average isoforms per gene, average transcript length (bp) and the proportion of monexonic genes (genes with only one exon) is reported for the Authors’ annotations and the OMAnnotator re-annotations.*

# References

[Robinson O, Dylus D, Dessimoz C. Phylo.io : Interactive Viewing and Comparison of Large Phylogenetic Trees on the Web. *Mol Biol Evol* 2016;**33**:2163–6.](https://www.zotero.org/google-docs/?RHpwoV)
